# Human IgE monoclonal antibodies define two unusual epitopes trapping dog allergen Can f 1 in different conformations

**DOI:** 10.1002/pro.70269

**Published:** 2025-08-19

**Authors:** Kriti Khatri, Alyssa Ball, Jill Glesner, Christina Linn, Lisa D. Vailes, Sabina Wünschmann, Scott A. Gabel, Jian Zhang, R. Stokes Peebles, Tomasz Borowski, Geoffrey A. Mueller, Martin D. Chapman, Scott A. Smith, Anna Pomés, Maksymilian Chruszcz

**Affiliations:** ^1^ Department of Biochemistry and Molecular Biology Michigan State University East Lansing Michigan USA; ^2^ Department of Chemistry and Biochemistry University of South Carolina Columbia South Carolina USA; ^3^ InBio Charlottesville Virginia USA; ^4^ National Institute of Environmental Health Sciences, Research Triangle Park North Carolina USA; ^5^ Vanderbilt University Medical Center Nashville Tennessee USA; ^6^ Jerzy Haber Institute of Catalysis and Surface Chemistry, Polish Academy of Sciences Kraków Poland

**Keywords:** Can f 1, conformational epitope, dog allergen, epitope mutants, human IgE antibody, IgE/allergen complexes, lipocalins

## Abstract

Molecular analysis of interactions between IgE antibody and allergen allows the structural basis of IgE recognition to be defined. Human IgE (hIgE) epitopes of respiratory lipocalin allergens, including Can f 1, remain elusive due to a lack of IgE‐allergen complexes. This study aims to map the structure of allergenic epitopes on Can f 1. The fragment antigen‐binding (Fab) regions of Can f 1 specific human IgE monoclonal antibodies (hIgE mAb) were used to determine the structures of IgE epitopes. Epitope mutants were designed to target Can f 1 epitopes. Immunoassays and a human FcεRI*α* transgenic mouse model of passive anaphylaxis in vivo were used to assess the functional activity of epitope mutants. Crystal structures of natural or recombinant Can f 1 complexed with two hIgE mAb 1J11 and 12F3 Fabs, respectively, were determined. The hIgE mAb bound to two partially overlapping epitopes and recognized two different Can f 1 conformations. The hIgE mAb 12F3 showed an unusual mode of binding by protruding its heavy chain CDR3 inside the Can f 1 calyx. Epitope mutants generated based on the structural analyses displayed a 64%–89% reduction in IgE antibody binding and failed to induce passive anaphylaxis in a human FcεRI*α* transgenic mouse model. In summary, the structures of Can f 1‐hIgE Fab complexes revealed two unique and partially overlapping epitopes on Can f 1. The modification of the identified IgE epitopes provides a pathway for the design of hypoallergens to treat dog allergies.

## 1 INTRODUCTION

Allergic disease caused by repeated exposure to cat and dog allergens affects 10%–20% of the world's population (Chan & Leung, 2018). Continued exposure to cat and dog allergens is one of the leading causes of asthma, which heavily impacts public health (Gao et al., 2020; Gergen et al., 2018; Ingram et al., 1995; Konradsen et al., 2014; van Hage et al., 2023). Specifically for dog allergens, more than 1 million increased asthma attacks have been estimated annually for the dog‐sensitized and exposed population of patients with asthma (Gergen et al., 2018). Eight dog (*Canis familiaris*) allergens are included in the World Health Organization and International Union of Immunological Societies Allergen Nomenclature database, among which Can f 1 is a major allergen (Pomés et al., 2018; Schou et al., 1991). More than 70% of the dog‐allergic population is allergic to Can f 1 and accounts for 50%–90% of IgE anti‐Can f 1 antibodies in dog‐allergic patients (Breitenbuecher et al., 2016; Konradsen et al., 2015). Like most mammalian respiratory allergens, Can f 1 belongs to the lipocalin protein family (Flower, 1996; Virtanen et al., 2012). Lipocalins share low sequence identity but have conserved structural motifs, characterized by an N‐terminal 3_10_ helix, a C‐terminal *α* helix, and a central calyx formed by two antiparallel *β*‐sheets that enclose a central hydrophobic cavity forming a *β*‐barrel for ligand binding (Dartt, 2011).

Allergen interactions with IgE antibodies are one of the key determinants of allergic symptoms in sensitized individuals (Galli & Tsai, 2012; Smith, Chruszcz, et al., 2023). IgE antibodies recognize specific binding sites (epitopes) on the allergen molecular surface, which are conformational in most inhalant allergens (Pomés, 2010; Pomés et al., 2020). The structure of the allergen bound to the antibody allows precise localization of residues that comprise the epitope, which enables a better understanding of the molecular basis of allergy and cross‐reactivity that can directly impact diagnostics, generation of avoidance guidelines, and therapeutic approaches (Pomés, 2010; Pomés et al., 2020, 2024).

Structurally identified IgE epitopes of lipocalin allergens, including Can f 1, remain elusive (Curin et al., 2017; Nakatsuji et al., 2022). Mapping of IgE epitopes on allergens has remained a big challenge due to the lack of allergen‐specific mAb. The recent development of hIgE mAb has overcome this hurdle using allergen‐specific IgE derived from B cells of symptomatic allergic patients (Smith, Reid Black, et al., 2023; Wurth et al., 2018). Here, we present the x‐ray crystal structures of Can f 1 in complex with hIgE mAb 1J11 and hIgE mAb 12F3. To our knowledge, the complex of natural Can f 1 (nCan f 1) with hIgE mAb 1J11 Fab is the first structure of an allergen isolated from a natural source bound to a hIgE mAb with the correct pairing of heavy and light chains as it occurs in vivo. The two hIgE mAb 1J11 and 12F3 recognized Can f 1 in two different conformational states, and 12F3 had a unique mode of interaction with the allergen. The structurally identified hIgE epitopes provided a coherent rationale for epitope‐based mutagenesis of Can f 1.

## 2 RESULTS

### 2.1 Can f 1 specific hIgE mAb

Four Can f 1 specific hIgE mAb were isolated from B cell hybridomas from allergic subjects (Table S.I, Supplementary Material). Among them, hIgE mAb 1J11 and 12F3 displayed high binding affinity to nCan f 1 with a dissociation constant (*K*_D_) of 0.2 ± 0.1 nM and 3.6 ± 1.3 nM, respectively, when measured by localized surface plasmon resonance (LSPR) (Table S.II). The variable gene sequences of 1J11 and 12F3 were moderately mutated versus the germline (Table S.III).

### 2.2 X‐ray crystal structures of Can f 1 and hIgE mAb fab complexes

Crystallization experiments were performed for hIgE mAb 1J11 and 12F3 with both nCan f 1 and rCan f 1 (recombinant Can f 1). X‐ray crystal structures were obtained for hIgE mAb 1J11 Fab complexed with nCan f 1 (nCan f 1‐1J11 Fab) and hIgE mAb 12F3 Fab complexed with rCan f 1 (rCan f 1‐12F3 Fab). Both hIgE mAb also crystallized in complex with a shorter recombinant Can f 1 construct, rCan f 1_C100S_ and the crystal structure of free rCan f 1_C100S_ was determined as well (Figures S.1–2, Supplementary Material). Details on these crystal structures and refinement statistics table (Table S.IV) are listed in Supplementary Material.

**FIGURE 1:**
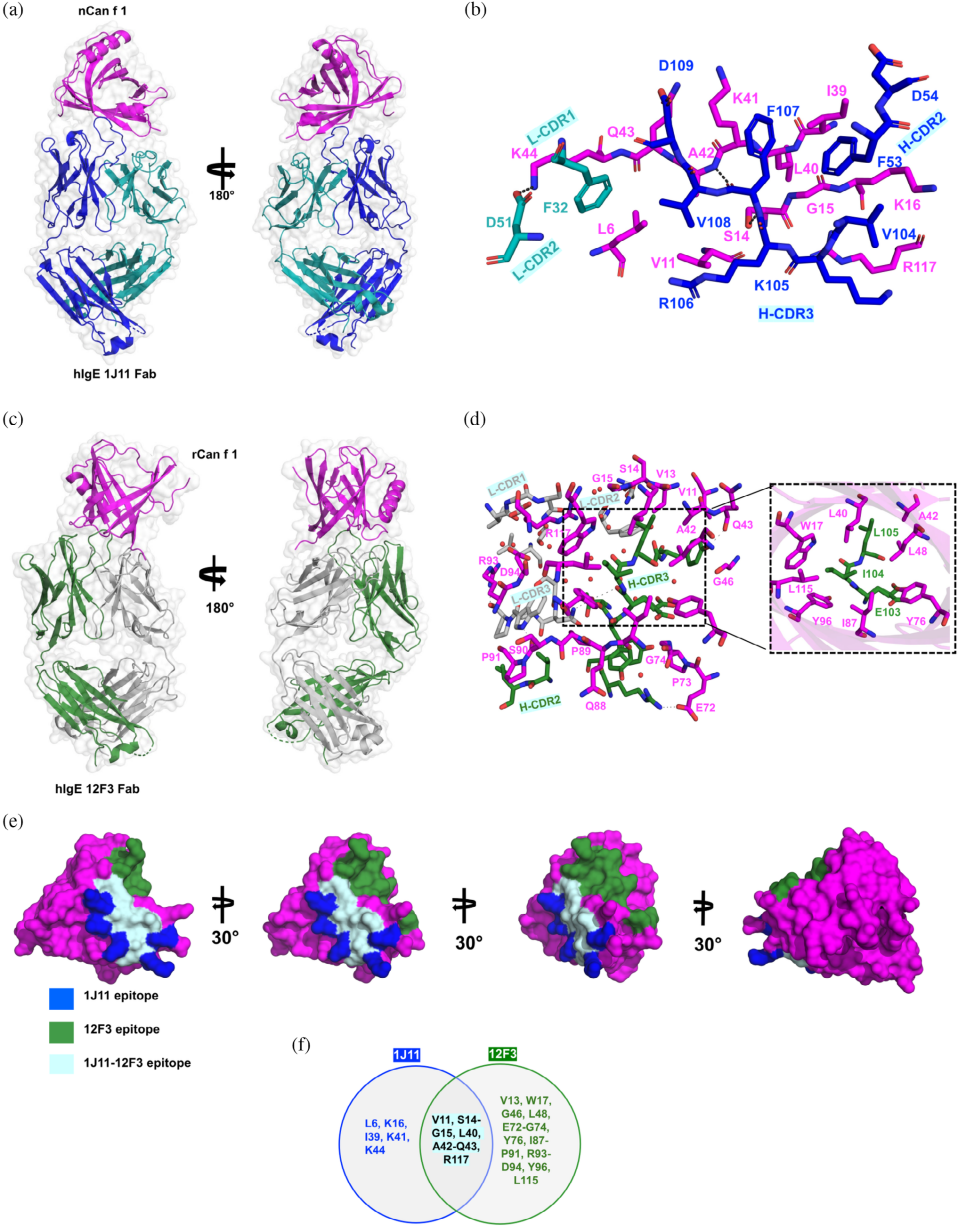
X‐ray crystal structures of Can f 1‐hIgE mAb Fab complexes. (a) Cartoon and space‐filling representations of the structure of nCan f 1(magenta) bound to hIgE mAb 1J11 Fab (heavy chain in blue and light chain teal green, respectively). (b) Interface between nCan f 1 and the 1J11 Fab showing the epitope‐forming residues of nCan f 1 (magenta) interacting with the CDRs of the heavy chain (blue) and light chain (teal) of 1J11 Fab in stick diagram. H‐bonds in the interface are shown in black dash. (c) Cartoon and space‐filling representations of the structure of rCan f 1 bound to hIgE mAb 12F3 Fab (heavy and light chains are shown in green and gray, respectively). (d) Interface of 12F3‐rCan f 1 showing epitope forming residues of rCan f 1 (magenta) and 12F3 antibody (green for heavy chain and gray for light chain) in stick diagram representation. Water molecules present in the interface are shown in red sphere and H‐bond interactions are shown in black dashes. (e) The epitopes of 1J11 (blue), 12F3 (green), and partially overlapping epitope for both IgE mAbs (pale cyan) are shown on the surface of Can f 1 and (f) Can f 1 residues forming epitopes of both IgE mAbs are shown in a Venn diagram.

The nCan f 1‐1J11 Fab complexes crystallized in P3_1_21 space group and diffracted to 3.1 Å (Figure 1a) (Table S.IV). In the elucidated structure of nCan f 1‐1J11 Fab, the total interface area between nCan f 1 and 1J11 Fab is 650 Å^2^ (Gao et al., 2020), of which a major fraction (72%) is contributed by the heavy chain of the antibody. The epitope region is defined by residues Leu6, Val11, Ser14‐Lys16, Ile39‐Lys44, and Arg117 (Figures 1b, S.1A). Of these epitope residues, Ser14, Lys16, Ala42, and Arg117 make five H‐bonds with the heavy chain, and Lys44 makes one H‐bond with the light chain of the 1J11 antibody (Table S.V). No crystal lattice contacts are observed in the epitope‐paratope region.

The complex of rCan f 1‐12F3 Fab crystallized in the P2_1_ space group and diffracted to 1.8 Å resolution (Figure 1c) (Table S.IV). The rCan f 1‐12F3 Fab complex reveals a large interface area of 1102 Å^2^ (Gao et al., 2020), of which 62% is contributed by the heavy chain and 38% is contributed by the light chain of the Fab. The interface displays a binding interaction between HCDR‐3 and rCan f 1 residues, in which the HCDR‐3 loop comprising residues Glu103, Ile104, and Leu105 inserts inside the *β*‐barrel of Can f 1 and makes non‐covalent interactions with the rCan f 1 residues Trp17, Leu40, Leu48, Tyr76, Ile87, and Tyr96 (Figure 1d). Altogether, 11 H‐bonds are formed in the interface of rCan f 1‐12F3 Fab, among which residues Gln43, Glu72, Tyr76, and Tyr96 make six H‐bonds with the heavy chain, and Ser14, Ser90, Arg93, and Tyr96 make five H‐bonds with the light chain of the antibody (Figure S.1B, Table S.VI). Crystal lattice contacts are not observed in the epitope‐paratope region. Both 1J11 and 12F3 Fabs recognized partially overlapping epitopes on Can f 1 formed by residues Val11, Ser14‐Gly15, Leu40, Ala42, Gln43, and Arg117 (Figure 1e). Interestingly, the Can f 1 residues Val11 and Ser14‐Gly15 that are recognized by both antibodies are in the protein fragment that has different conformations in the structures we determined.

### 2.3 hIgE mAb recognize two different conformations of Can f 1

**FIGURE 2:**
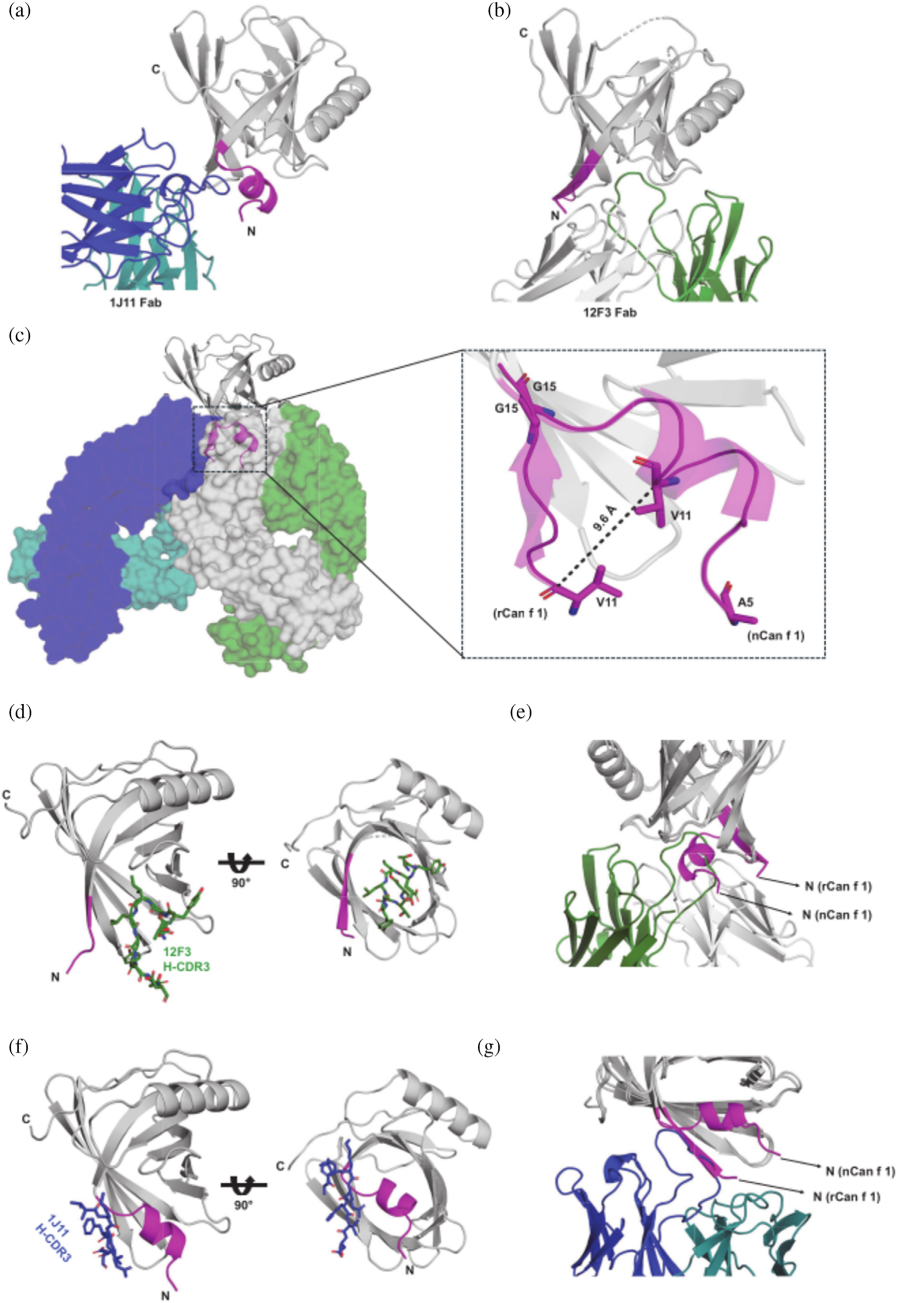
hIgE recognizes different conformational Can f 1N‐terminus. HIgE 1J11 binding to nCan f 1 (a) and hIgE 12F3 binding to rCan f 1 (b) shows the changed orientation of N‐terminus (shown in magenta) in each structure. (c) Superimposition of hIgE mAb 12F3 and 1J11 complexes with Can f 1 shows the orientation of the N‐termini and the distance between the residue Val11 in Can f 1 within respective complexes with hIgE mAb 12F3 and 1J11 Fab. (d) The 12F3 H‐CDR3 insertion inside the hydrophobic cavity of rCan f 1. (e) Superimposition of nCan f 1 in the 12F3‐rCan f 1 structure shows that the N‐terminus conformation as observed in nCan f 1‐1J11 interface would hinder the 12F3‐HCDR‐3 binding inside the Can f 1 cavity. (f) 1J11 H‐CDR3 binding orientation in nCan f 1 interface. (g) Superimposition of rCan f 1 in the 1J11‐nCan f 1 structure shows that the N‐terminus conformation of rCan f 1 from 12F3‐rCan f 1 hinder the 1J11 H‐CDR3 binding to Can f 1.

Comparison of Can f 1 structures in complexes with 1J11 and 12F3 Fab revealed that the residues Val11‐Gly15 of Can f 1 adopt different conformations (Figure 2a–c). Very interestingly, 12F3 recognizes a Can f 1 conformation in which a shift of the N‐terminus residues away from blocking the opening of the *β*‐barrel allows the 12F3 HCDR‐3 to access and interact with residues located inside the *β*‐barrel (Figure 2d). Structurally, it is evident that such orientation and interactions would not be possible if the N‐terminus had the conformation as the one observed for the Can f 1 in complex with 1J11, where the N‐terminus blocks access to the cavity (Figure 2e). Similarly, based on the nCan f 1‐1J11 Fab crystal structure, most likely the 1J11 Fab would not bind to the allergen in the conformation that is recognized by the 12F3 Fab (Figure 2f, g).

To further inspect whether the conformational change of the N‐terminus is also observed in other Can f 1 structures, available experimental models of Can f 1, either bound with hIgE mAb 1J11 (PDB: 8VQF) and 12F3 (PDB: 9NPI) or unbound (PDB: 7DRU, 8EPU, 8VQG) were compared (Min et al., 2023; Nakatsuji et al., 2022). Interestingly, among these structures, only the nCan f 1 within the nCan f 1‐1J11 Fab complex structure provides a more complete model of the N‐terminus, starting at residue Ala5 and 3_10_ helix formed by Lys8 to Val13. Structural comparison reveals that unbound Can f 1 structures exhibit a similar N‐terminus conformation as the one observed in the nCan f 1 from the nCan f 1‐1J11 complex (Figure S.3). Only when complexed with 12F3 does the N‐terminus of rCan f 1 have a different conformation. Similarly, the C‐terminus of Can f 1 (PDB: 8EPU) differs in conformation when compared to the rest of the Can f 1 structures (Figure S.3). These structures provide evidence that, while the overall structural fold is similar, both N and C‐termini of Can f 1 are flexible and can undergo conformational changes.

To investigate the flexible nature of the N‐terminus of Can f 1, a molecular dynamics (MD) simulation experiment was performed. Results of MD simulations performed for Can f 1 indicate that the conformation of the N‐terminus region of the protein is very fluid (Figure S.4A) and it oscillates between the predominant helical and a less abundant extended conformation (Figure S.4B–D). Snapshots of three independent MD trajectories, each spanning 1200 ns, were clustered based on RMSD for backbone atoms of Ala5‐Gly15 N‐terminus fragment, and the representative structures of the largest clusters were superimposed (Figure S.5). This analysis shows the N‐terminus fragment at different stages of unwinding the helix.

### 2.4 Structural basis for designing hIgE epitope mutants of Can f 1

The epitope analysis of the nCan f 1‐1J11 Fab and rCan f 1‐12F3 Fab structures for the first time provided a structure‐based rationale to design hIgE epitope mutants of Can f 1. Initially, six epitope mutants (V11K, G15K, K16A, K44E, G15K‐K16A (KA) and G15K‐K16A‐K44E (KAE)) targeting the 1J11 epitope (single epitope mutants) were designed and produced. Later, Can f 1 mutants targeting simultaneously the epitopes for the two hIgE mAb 1J11 and 12F3 (double epitope mutants) were designed based on structural insights from nCan f 1‐1J11 Fab and rCan f 1‐12F3 Fab structures. Both IgE mAb 1J11 and 12F3 interact with Can f 1 N‐terminal residues, and immunoassays showed that mutant KA was effective at reducing IgE mAb 1J11 binding (Figure S.6). Hence, mutations of residues S14A‐G15K or S14A‐G15K‐K16A were used to design three double epitope mutants: (a) AKAA comprising the amino acid substitutions S14A‐G15K‐D94A‐Y96A, (b) AKGA comprising S14A‐G15K‐P89G‐Y96A, and (c) AKA‐AAA comprising S14A‐G15K‐K16A‐R93A‐D94A‐Y96A. These mutants were designed to reduce binding of both hIgE mAb 1J11 and 12F3 to Can f 1. Details on the design of epitope mutants are in Supplementary Material.

### 2.5 Reduced binding of hIgE mAb 1J11 and 12F3 to epitope mutants

**FIGURE 3:**
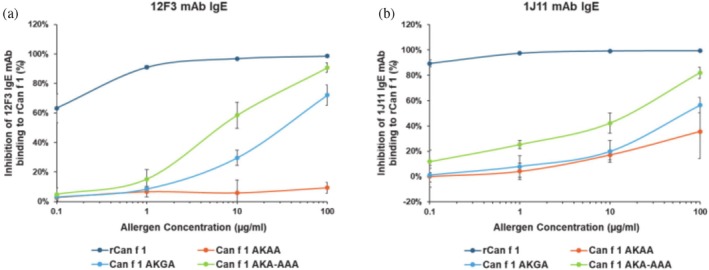
Inhibition Immunoassays of double epitope mutants. Inhibition immunoassays of 1J11‐12F3 double epitope mutants show the reduced ability of epitope mutants to inhibit (a) hIgE mAb 12F3 and (b) hIgE mAb 1J11 binding to wildtype Can f 1. The least inhibition of mAb binding to rCan f 1 was observed for mutant AKAA, followed by AKGA and AKA‐AAA as compared to wildtype Can f 1. The lowest percentage of inhibition exhibited by mutant AKAA implies poor interaction of this mutant with both antibodies arising from disrupted epitopes specific to these hIgE mAb.

The ability of epitope mutants to inhibit binding of hIgE mAb 12F3 or 1J11 to wildtype Can f 1 was assessed via inhibition immunoassays. All three double epitope mutants showed decreased inhibition of 1J11 and 12F3 binding to wildtype rCan f 1, among which mutant AKAA showed the largest decrease (64% for 1J11 and 89% for 12F3), at the maximum concentration of inhibitor tested (100 μg/mL) (Figure 3). The decrease in inhibition observed for mutant AKAA suggests that both mAb poorly recognized the mutant in comparison to the wildtype Can f 1. In agreement with the inhibition assays, all double epitope mutants were unable to bind both 1J11 and 12F3 as designed, while being able to bind the control hIgE mAb 10G1 and 4F5 in a dose‐dependent manner in direct binding immunoassays (data not shown). Similarly, all single epitope mutants except for V11K exhibited reduced binding to hIgE mAb 1J11 but comparable binding versus wildtype Can f 1 to hIgE mAb 12F3 and 4F5 (Figure S.6B–D). The binding of the hIgE mAb 12F3 to the single 1J11 epitope mutant indicates that even though the epitopes of 1J11 and 12F3 partially overlap, disrupting the 1J11 epitope alone does not prevent 12F3 binding. Similarly, binding of all (single and double) epitope mutants to control IgE mAb 4F5 and 10G1 confirms the retention of non‐overlapping IgE epitopes and proper folding of the mutants. The combination of S14A‐G15K‐D94A‐Y96A mutations was most effective in decreasing hIgE binding to both 1J11 and 12F3 epitopes; hence, mutant AKAA was used for further experiments.

### 2.6 Impact of 1J11‐12F3 epitope mutation on polyclonal IgE response

**FIGURE 4:**
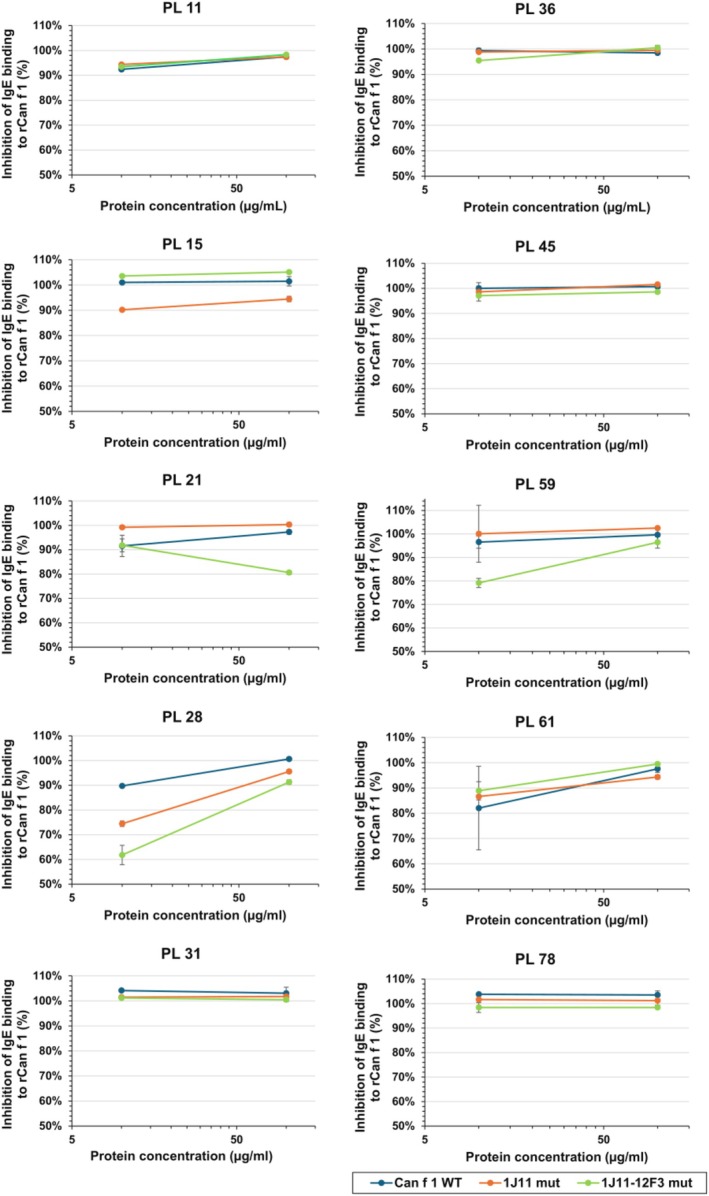
Inhibition of polyclonal antibody (pAb) IgE binding to rCan f 1 by wildtype and mutant rCan f 1. The inhibition of polyclonal IgE from 10 dog allergic patient sera (represented by “PL”) by wildtype and mutant Can f 1. Lowered inhibition of pAb binding to rCan f 1 is shown for PL‐15 and PL‐28 for 1J11 mutant KA, and PL‐21, PL‐28, and PL‐59 for 1J11‐12F3 mutant AKAA. Data are averages of duplicates ± SD.

The ability of mutant KA and AKAA to inhibit the IgE response from dog allergic patients to wildtype Can f 1 was assessed by immunoassays (Figure 4) using plasma from 10 Can f 1 allergic subjects. A small decrease in inhibition was observed in two and three out of ten tested plasmas for mutants KA (PL‐15 and PL‐28) and AKAA (PL‐21, PL‐28, and PL‐59), respectively (Figure 4). Overall, both mutants showed strong inhibition of IgE binding to wildtype Can f 1.

### 2.7 Epitope mutant reduced passive anaphylaxis in transgenic mouse‐model

The single epitope mutant KA and the double epitope mutant AKAA were tested for their ability to crosslink hIgE mAb in vivo to induce mast cell degranulation and passive systemic anaphylaxis in a mouse model. Human Fc*ε*RI*α* transgenic mice were sensitized with two or three Can f 1‐specific hIgE mAb prior to challenging with wildtype or epitope mutant Can f 1. Simultaneous cross‐linking of at least two IgE mAb binding to non‐overlapping epitopes would be required for systemic anaphylaxis to occur, given the monomeric nature of Can f 1 (as observed in the complex structures). Therefore, hIgE mAb pairs that bind to non‐overlapping epitopes on the allergen were selected for mice IgE sensitization. Anaphylaxis was indicated by the reduction of the core body temperature of the mice when challenged with Can f 1.

**FIGURE 5:**
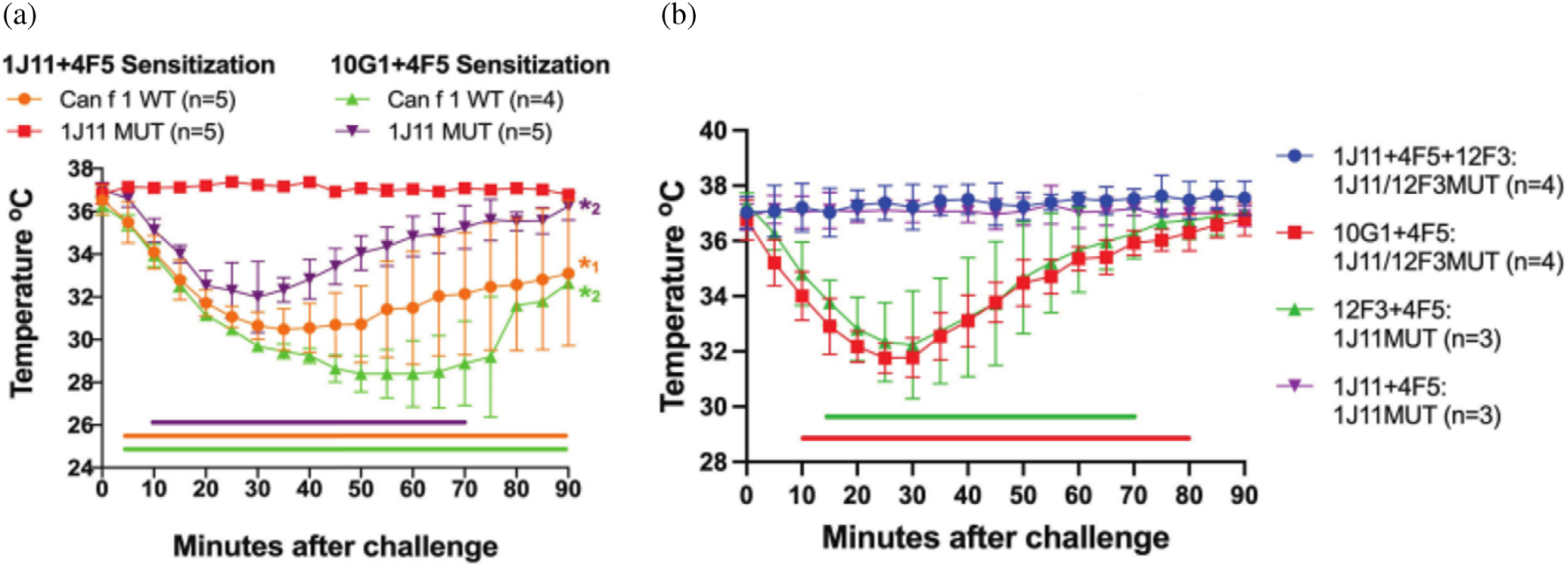
Murine model of passive systemic anaphylaxis. (a) Human FcεRI*α* transgenic mice sensitized with 100 μg total of Can f 1 specific IgE mAb 1J11 + 4F5 or 10G1 + 4F5 were challenged with 50 μg of rCan f 1 (Can f 1 WT) or 1J11 single epitope mutant KA (1J11 MUT). (b) Mice sensitized with 100 μg total of Can f 1 specific IgE mAb 1J11 + 4F5 + 12F3, 10G1 + 4F5, 12F3 + 4F5 or 1J11 + 4F5, and challenged with 1J11‐12F3 double epitope mutant AKAA (1J11/12F3 MUT) or 1J11 single epitope mutant KA (1J11 MUT), respectively. Anaphylaxis was monitored using an implanted temperature probe for 90 min following the challenge. Time points with calculated *p*‐values <0.05 are underscored with a colored bar. Data are means ± SD of each experimental group. The number of mice (*n*) for each experimental group is shown in parenthesis. The number of mice in each group that succumb to anaphylaxis is indicated by an asterisk.

Mice were sensitized with two or three hIgE mAb: (a) 1J11‐12F3‐4F5, (b) 10G1‐4F5, (c) 1J11‐4F5, and (d) 12F3‐4F5 prior to challenging them with wildtype or mutant Can f 1. Mice sensitized with control hIgE mAb pair 10G1‐4F5 exhibited anaphylaxis when challenged with wildtype rCan f 1, 1J11 mutant KA, and 1J11‐12F3 mutant AKAA (Figure 5a, b). These results additionally confirm that the epitopes for mAb 10G1 and 4F5 are preserved in both mutants. Anaphylaxis also occurred in mice sensitized with (a) hIgE mAb pairs 1J11‐4F5 and 12F3‐4F5 when challenged with wildtype rCan f 1 (Figure 5a), and (b) hIgE mAb pair 12F3‐4F5 when challenged with 1J11 epitope mutant KA, which retains both 12F3 and 4F5 epitopes (Figure 5b).

When mice were sensitized with two or three hIgE mAb: (a) 1J11‐4F5, and (b) 1J11‐12F3‐4F5, and challenged with the single 1J11 epitope mutant KA and the double 1J11‐12F3 epitope mutant AKAA, respectively, no anaphylaxis was observed (Figure 5a, b). This result indicates that IgE mAb 1J11 or 12F3 did not cross‐link with IgE mAb 4F5 due to their poor interaction with mutant allergens and failed to induce anaphylaxis.

## 3 DISCUSSION

Here, we report the first structures of allergenic epitopes recognized by hIgE mAb directed against the major dog allergen, Can f 1. The nCan f 1‐1J11 Fab is the first published structure of a dog allergen purified from a natural source in complex with human IgE Fab. The structures revealed previously uncharacterized conformational IgE epitopes on Can f 1 and represent the allergen‐antibody interactions as they occur in vivo. Unlike linear B cell epitopes that can be found in any part of the allergen, especially in foods due to digestion or processing, conformational epitopes are more common in aeroallergens such as Can f 1 and are located on the allergen molecular surface. In contrast, the complex rCan f 1‐12F3 Fab proved to be an exception in that the unique IgE epitope was located inside the *β*‐barrel of Can f 1. This type of allergen‐antibody interaction has not been reported before.

Analyses of Can f 1 in complex with hIgE mAb 1J11 or 12F3 Fabs showed that the N‐terminal residues of the allergen contribute to partially overlapping epitopes recognized by both antibodies. More interestingly, these N‐termini displayed conformational changes within the structures of nCan f 1‐1J11 and rCan f 1‐12F3 complexes such that both hIgE mAb recognize two different conformations of the allergen. This is consistent with our MD results that indicate the presence of different conformational states of the Can f 1 N‐terminus. These findings present an unusual case of IgE recognizing different conformational states of the same allergen. The biological significance of the hIgE mAb epitopes was established by designing 1J11‐12F3 epitope mutants of Can f 1, which showed reduced capacity to bind both 1J11 and 12F3 mAb, as well as the inability to induce passive anaphylaxis in a transgenic mouse model. However, when tested with pAb in patient plasma, the mutants' recognition was comparable to wildtype Can f 1. These results imply that the 1J11 and 12F3 epitopes are not immunodominant for the tested plasma. We speculate that this finding may be related to the fact that the hIgE mAb 12F3 recognizes the residues from the internal Can f 1 cavity instead of the usual solvent‐accessible epitopes present on the molecular surface or the possibility that immunodominant epitopes are found on more rigid fragments of allergens/antigens.

Previously, conformation‐specific IgG Fabs have been identified using phage display technology for catalytically competent “on” and catalytically inactive “off” conformers of caspase‐1 (Gao et al., 2009). Similarly, neutralizing IgG antibodies that target the HIV‐1 envelope glycoprotein across different conformational states have been reported (Ozorowski et al., 2017). However, there has not been structural evidence of IgE mAb recognizing different conformational states of the same allergen.

The role of the conformational state and structural flexibility of antibodies and antigens has been well reported; although discrepancies remain regarding whether the antibody prefers antigen binding via conformational selection or induced fit model and whether antibodies target conformationally rigid or flexible epitopes for immune recognition (Berger et al., 1999; Blackler et al., 2022; Fernández‐Quintero et al., 2019; Galanti et al., 2016; Guo et al., 2024; Keskin, 2007; Nussinov et al., 2014; Rini et al., 1992; Tengerdy & Faust, 1968). In the case of allergens, it was reported that structurally flexible isoforms of Bet v 1 and Cor a 1 had lower IgE binding than the rigid isoforms of these two allergens (Führer et al., 2021). On the other hand, the structural flexibility of grass pollen allergen Phl p 5 enhanced IgE crosslinking, and consequently its allergenic activity (Göbl et al., 2017). The conformational flexibility of an antigen is also related to the immunodominance of its epitopes, as some studies suggest that the immunodominant regions on the antigen surface are generally less flexible (Cook et al., 2022; Dall'antonia et al., 2014; Marini‐Rapoport et al., 2024; Meola et al., 2015). This is consistent with our study, as we showed that both hIgE mAb 1J11 and 12F3 bind to the flexible N‐terminal part of the allergen and the identified epitopes were not immunodominant among the tested patient plasma.

A potential explanation is that the conformational states that were trapped by hIgE mAb 1J11 and 12F3 are related to the physiological function of Can f 1. It was reported that lipocalins (including Can f 1) transfer fatty acids and/or hydrophobic molecules by binding them in their hydrophobic cavities inside the *β*‐barrel (Flower, 1996; Stopková et al., 2021). The N‐terminus of Can f 1 is at the entrance of the *β*‐barrel and may play a role in opening the access to the ligand binding cavity. This is supported by the results of our MD simulations, as well as crystal structure analysis of bound and free forms of Can f 1. These findings suggest that conformational flexibility is also important for the physiological function of Can f 1 homologs. However, none of the Can f 1 structures (conformationally different) including nCan f 1, showed the presence of ligands (fatty acids) in their cavity, and the physiological ligand of Can f 1 is still unknown. Therefore, it is not known how ligand binding will impact the binding of these IgE mAb to Can f 1.

In summary, the results have defined the molecular and conformational state of Can f 1 as it relates to binding to human IgE mAb. The use of hIgE mAb provided precise localization of specific IgE epitopes that are found in individuals with dog allergy. The increasing prevalence of dog allergy worldwide and the risk of IgE sensitization to dog allergens emphasize the need for efficacious allergy diagnostic and allergy management strategies (Dávila et al., 2018). Mutants with disrupted hIgE mAb epitopes may have potential therapeutic applications for dog allergy immunotherapy. The results also highlight the need to identify additional Can f 1 IgE epitopes to better understand the molecular interactions involved in IgE/allergen recognition.

## 4 MATERIALS AND METHODS

### 4.1 Generation of Can f 1‐specific hIgE mAb

The hIgE mAb were isolated from allergic subjects and then screened for Can f 1, as described previously (Ball et al., 2024; Khatri et al., 2022; Wurth et al., 2018). The binding affinity of hIgE mAb 1J11 and 12F3 for Can f 1 was assessed by LSPR using an OpenSPR (Nicoya Lifesciences, Kitchener, ON, Canada). The hIgE mAb 1J11 and 12F3 were expressed as Fab in CHO cells and used for binding studies with Can f 1. Details of antibody isolation, production, and purification are provided in Supplementary material.

### 4.2 Production of Can f 1 and epitope mutants

The nCan f 1 was purified from dog fur extract by mAb affinity chromatography, and a purity of approximately 95% was assessed by silver‐stained SDS‐PAGE. Two constructs of recombinant Can f 1 were used for this study: (a) rCan f 1 with full‐length mature protein sequence ranging from Glu1 to Gln156 at the C‐terminus and (b) rCan f 1_C100S_ construct, which has a shorter N terminal sequence spanning from Asp9 in the N‐terminus to Gln156 in the C‐terminus with a Cys100Ser mutation (Min et al., 2023). DNA constructs coding for epitope mutants were either commercially purchased or designed via site‐directed mutagenesis. Both wildtype and mutant Can f 1 allergens were expressed in *Escherichia coli*, purified using affinity chromatography and gel filtration, and their folding was assessed by 1D‐NMR (Figures S.7, 8). Details on Can f 1 production and purification are provided in Supplementary Material.

### 4.3 Crystallizations and structural studies of Can f 1‐hIgE Fab complexes

Purified nCan f 1 and rCan f 1(rCan f 1 and rCan f 1_C100S_) were mixed with hIgE mAbs 1J11 and/or 12F3 Fab in a 1:1 molar ratio, purified using gel filtration, and used for crystallization experiments. Details on crystallization, structure determination, and computational approaches are provided in Supplementary material.

### 4.4 Immunoassays to measure antibody‐allergen interactions

Antibody binding to the allergens was assessed by: (a) direct binding of hIgE mAb to rCan f 1 or epitope mutants coating the plate, and (b) inhibition assays preincubating rCan f 1 or epitope mutants with hIgE mAb or polyclonal antibodies (pAbs) before measuring antibody binding to the rCan f 1 coating the plate. Comparable inhibition of IgE mAb binding to Can f 1 was observed when plates were coated with either nCan f 1 or rCan f 1 (data not shown). Therefore, subsequnet immunoassay experiments were carried out using rCan f 1. Can f 1 specific IgE mAb 4F5 and 10G1, which recognize epitopes non‐overlapping with hIgE mAb 1J11 or 12F3, were used as controls. Can f 1 specific IgE levels in the 10 patients' plasma were measured by the ImmunoCAP system (Table S.VII). Informed donor consent was obtained from each individual prior to the first donation. For details on immunoassay experiments, see Supplementary material.

### 4.5 Assessment of the capacity to cross‐link IgE in vivo using a mouse model of passive anaphylaxis

Mouse studies were carried out in accordance with recommendations in the Guide for the Care and Use of Laboratory Animals of the National Institutes of Health. Human FcεRI*α* transgenic mice were sensitized with two or three IgE mAb (e.g., 1J11 and 4F5) via i.v. injection (100 μg total) 2 days prior to challenging with rCan f 1 (50 μg wildtype or mutant) by i.p. injection. The decrease in core body temperature of the mice after being challenged with the allergen was recorded with an implanted temperature probe. Supplementary material contains additional details of the in vivo experiments.

## AUTHOR CONTRIBUTIONS

**Kriti Khatri:** Investigation; methodology; visualization; formal analysis; writing – original draft; writing – review and editing. **Alyssa Ball:** Investigation; writing – review and editing; visualization; formal analysis; methodology. **Jill Glesner:** Investigation; visualization; writing – review and editing; formal analysis; methodology. **Christina Linn:** Investigation; writing – review and editing; formal analysis. **Lisa D. Vailes:** Investigation; writing – review and editing. **Sabina Wünschmann:** Investigation; writing – review and editing; formal analysis. **Scott A. Gabel:** Investigation; visualization; formal analysis. **Jian Zhang:** Investigation; writing – review and editing; methodology; visualization; formal analysis. **R. Stokes Peebles Jr.:** Investigation; writing – review and editing; formal analysis. **Tomasz Borowski:** Investigation; writing – original draft; writing – review and editing; formal analysis; visualization. **Geoffrey A. Mueller:** Investigation; writing – review and editing; formal analysis. **Martin D. Chapman:** Conceptualization; funding acquisition; writing – review and editing; validation; formal analysis; supervision. **Scott A. Smith:** Conceptualization; funding acquisition; writing – original draft; writing – review and editing; visualization; methodology; formal analysis; supervision. **Anna Pomés:** Conceptualization; funding acquisition; writing – original draft; methodology; writing – review and editing; formal analysis; supervision; visualization. **Maksymilian Chruszcz:** Conceptualization; funding acquisition; writing – original draft; methodology; writing – review and editing; visualization; formal analysis; supervision.

## Supporting information


Data S1

## Data Availability

The data that support the findings of this study are available from the corresponding author upon reasonable request.
