# Supplementary material for: Human IgE monoclonal antibodies define two unusual epitopes trapping dog allergen Can f 1 in different conformations
Source: Protein Sci. 2025 Aug 19;34(9):e70269. doi: 10.1002/pro.70269 (PMC12363406; doi:10.1002/pro.70269)
Supplement: Supplementary file 1 — Data S1 [file PRO-34-e70269-s001.docx]

**Supplementary Materials**

**Human IgE monoclonal antibodies define two unusual epitopes trapping dog allergen Can f 1 in different conformations**

Kriti Khatri^1,2^, Alyssa Ball^3^, Jill Glesner^3^, Christina Linn^1^, Lisa D. Vailes^3^, Sabina Wünschmann^3^, Scott A. Gabel^4^, Jian Zhang^6^, R. Stokes Peebles Jr.^6^, Tomasz Borowski^5^, Geoffrey A. Mueller^4^, Martin D. Chapman^3^, Scott A. Smith^6^, Anna Pomés^3*^, Maksymilian Chruszcz^1,2*^

Affiliations:

^1^ Michigan State University, East Lansing, MI, USA

^2^ University of South Carolina, Columbia, SC, USA

^3^ InBio, Charlottesville, VA, USA

^4^ National Institute of Environmental Health Sciences, Research Triangle Park, NC, USA

^5^ Jerzy Haber Institute of Catalysis and Surface Chemistry, Polish Academy of Sciences, Kraków, Poland

^6^ Vanderbilt University Medical Center, Nashville, TN, USA

*Corresponding authors:

Maksymilian Chruszcz

Michigan State University

603 Wilson Rd,

East Lansing, MI 48824

chruszcz@msu.edu

Anna Pomés

InBio

700 Harris Street

Charlottesville, VA 22903

apomes@inbio.com

Page count: 35

Word count: 3,792

This file includes:

- Supplementary Methods
- Supplementary Results
- Supplementary Figures; S.1-S.8
- Supplementary Tables; S.I-IX
- References

**Supplementary Methods:**

**Generation of human hybridomas expressing IgE mAbs**

Allergic subjects were identified and recruited during a routine visit with their allergist provider. Blood samples were collected as approved by the Vanderbilt University Medical Center Institutional Review Board (IRB 141330 and 142030) (Table S.I). Patient Astarte 180 PBMCs were purchased from Astarte Biologics LLC (Cat # 1007, Lot #782AP11). IgE secreting human hybridomas were generated using methodology that has been described previously in detail.^1^ Cryopreserved peripheral blood mononuclear cells (PBMCs) samples were grown for seven days prior to screening culture supernatant for IgE secretion by ELISA. Cells within wells containing IgE measurements greater than 5 times that of background were selected for immortalization. No antigen specificity was assessed at this point in the generation of human IgE monoclonal antibodies (hIgE mAbs). The nonsecreting myeloma cell line MFP-2 (ATCC HB-12482) was fused with B cells using electrical cytofusion to generate human hybridomas. Cytofusion was performed using a BTX ECM 2001 generator and hybridomas were selected in hypoxanthine-aminopterin-thymidine (HAT) medium containing ouabain for fourteen days. Wells containing hybridomas secreting IgE antibody then were cloned biologically by indexed single cell flow cytometric sorting into 384-well culture plates. Each hybridoma then was expanded for large-scale expression in serum free medium (Gibco Hybridoma-SFM; Invitrogen, 12045084). IgE antibody then was purified by immunoaffinity chromatography (Omalizumab covalently coupled to GE Healthcare NHS activated HiTRAP; 17-0717-01) and used for determination of antigen specificity and all subsequent characterizations.

**Affinity measurements by localized Surface Plasmon Resonance**

The binding affinity of hIgE mAbs (1J11 and 12F3) to nCan f 1 was measured by LSPR using an OpenSPR^TM^ (Nicoya Lifesciences, Kitchener, ON, Canada) instrument. To measure the affinity (K_D_), the hIgE mAb 1J11 or 12F3 were immobilized on a biotin sensor via the biotin-streptavidin kit and biotinylated ChromoTek Nano-CaptureLigand™ human IgE, VHH (ProteinTech, Rosemont, IL, USA) and nCan f 1 was flowed over with running buffer (1x PBS, 0.5% Tween20, 100 mM NaCl) at concentrations from lowest to highest 0, 3.1, 9.26, 27.28, 83.33, or 250 nM. TraceDrawer® software was used to perform the analysis of the OpenSPR^TM^ data and calculate the K_D_.

**RNA isolation, RACE sequencing, and plasmid construction**

Cell pellet for 1J11 and 12F3 mAbs were generated from respective 1 million clonal IgE-expressing human hybridoma cells as previously described.^1^ Total RNA extraction was performed with RNeasy Mini Kit (Qiagen, Germantown, MD, USA). Reverse transcription PCR (RT-PCR) to synthesize 1J11 cDNA from total RNA was performed. 5’ RACE using the cDNA product was done with primers designed in the constant antibody region with separate PCR reactions for antibody heavy and light chains. Following PCR, cloning was done with IN-Fusion HD Cloning Kit (Takara, Mountain View, CA, USA). Similarly, for 12F3 cDNA synthesis, RT-PCR was performed for 30 cycles with a 5′ primer set described previously and a 3′ primer specific to the IgE constant region using OneStep RT-PCR kit (Qiagen: 210210).^2^ Following gel purification, 12F3 cDNA product was cloned into pCR2.1 using a TA cloning kit (Invitrogen: 45-0046). Both antibody genes were isolated by plasmid preparation and Sanger sequenced. Variable regions were determined by analysis using IMGT/V-QUEST database.

**Expression and purification of 1J11 and 12F3 Fabs**

The DNA encoding for the 1J11 and 12F3 Fabs were synthesized by GeneArt (Regensburg, Germany) with codon optimization for expression in CHO cells. DNA fragments containing either the Fab heavy or light chains were then cloned separately into a pcDNA3.4 vector (Invitrogen, San Diageo, CA, USA) by TOPO cloning. Endotoxin-free transfection grade plasmid purification was performed with a PureLink MidiPrep Kit (Life Technologies, Carlsbad, CA, USA). Plasmid quantification was done with UV spectroscopy (260 nm). Plasmids containing either antibody heavy or the light chain sequences were filtered with a 0.2 µm spin filter (Corning, Tewksbury, MA, USA). Plasmid DNA was combined with ExpiFectimine ^TM^ (Thermo Fisher, Waltham, MA) for complexation reaction. The antibody containing plasmid DNA was transfected in a 1:1 ratio of heavy to light chains into ExpiCHO-S^TM^ cells (Thermo Fisher, Waltham, MA, USA) in suspension. 24 hours (hrs) after transfection the cells were fed, and enhancer was added. Cells were cultured for 13 days in ExpiCHO media (Thermo Fisher, Waltham, MA, USA) at 37°C, 8% CO_2_, secreting recombinant Fab into the cell supernatant. Antibody containing cell culture supernatant was clarified by centrifugation at 2,000 x g for 30 minutes and filtered with a 0.2 µm vacuum filter (Corning, Tewksbury, MA, USA).

Purification of the recombinant Fabs (1J11 or 12F3) was performed by nickel affinity with a HisTrap^TM^ HP prepacked column (Cytivia, Marlborough, MA, USA) used according to manufactures instructions with an AKTA Pure system (Cytivia, Marlborough, MA, USA). Fractions containing detectable 1J11 or 12F3 Fab, by measuring absorbance at 280 nm, were combined. Purity of the Fabs was assessed by Coomassie stained SDS-PAGE.

**Expression and purification of Can f 1 and epitope mutants**

Natural Can f 1 was purified from dog fur extract by mAb affinity chromatography using approach similar to described previously.^3^ The DNA construct encoding for rCan f 1_C100S_, starting from residue Asp9 to Gln156 with C100S mutation was expressed as explained previously.^4^ The synthetic DNA constructs of Can f 1 double epitope mutants AKAA (Ser14Ala-Gly15Lys-Asp94Ala-Tyr96Ala), AKGA (Ser14Ala-Gly15Lys-Pro89Gly-Tyr96Ala) and AKA-AAA (Ser14Ala-Gly15Lys-Lys16A-Arg93Ala-Asp94Ala-Tyr96Ala) and single epitope mutants (G15K, K16A, K44E, G15K-K16A-K44E (KAE)) were harbored in pET-28a (+) vector with N-terminal 6xHis-tag followed by Tobacco Etch Virus (TEV) protease cleavage site and mature protein sequence starting from Glu1 to Gln156 at C terminal were obtained from SynBio Technologies (Monmouth Junction, New Jersey, USA). Site-directed mutagenesis was performed to design full-length rCan f 1 from mutant G15K-K16A (KA), mutant KA from mutant K16A and mutant V11K from rCan f 1_C100S_ DNA constructs (Table S.VIII).

The wildtype or mutant Can f 1 plasmid was transformed in *Escherichia coli* BL-21 (DE3) and cultured in a liter Luria-broth (LB) media supplemented with 10% glycerol and 100 μg/mL antibiotics of choice. Cultures were grown shaking at 150 rpm at 37°C until the OD of 0.8, then cooled to 16°C before inducing with 0.4 mM isopropyl β-D-1-thiogalactopyranoside (IPTG). After induction, cultures were grown overnight and harvested by centrifugation in a Beckman Coulter Ultracentrifuge at 4°C at 9,000 g for 10 min. For the purification, cell pellets were lysed using buffer (50 mM Tris, 500 mM NaCl, 10 mM imidazole, pH 7.5), sonicated in Branson Sonifier 450, and centrifuged in Beckmann Coulter Ultracentrifuge at 35,000 g for 1 hour (h) at 4°C. The supernatant collected after centrifuge was purified by affinity chromatography using Ni-NTA resin (ThermoFisher Scientific) in a Bio-Rad column (Hercules, CA). The N-terminus 6xHis purification tag was cleaved by incubating purified protein with TEV protease at room temperature for 4 h or overnight at 4°C. The cleaved proteins were purified via a Superdex-10/300 column attached to an AKTA Pure FPLC system (GE Healthcare, Chicago, IL, USA). Elution fractions corresponding to the chromatogram peaks were pooled together, concentrated, and used for X-ray crystallography and required experiments.

**Can f 1 complexes with hIgE 1J11 and 12F3 mAb Fab**

Purified Can f 1 (natural or recombinant) was mixed with hIgE mAbs 1J11 or 12F3 Fab in a 1:1 molar ratio and ran in a Superdex-10/300 attached to an AKTA Pure FPLC system (GE Healthcare, Chicago, IL, USA) equilibrated with 50 mM Tris, 150 mM NaCl, pH 7.5. The fractions corresponding to the Can f 1-hIgE complexes were pooled together and concentrated before setting crystallization experiments.

**Crystallization, data collection, and structure determination of Can f 1- hIgE Fab complexes and Can f 1**

Crystallization experiments were performed using sitting drop vapor diffusion method using commercial crystallization screens at room temperature as well as 277 K. Diffracting crystals were collected from various crystallization conditions (Table S.IX) and flash frozen in liquid nitrogen before data collection. Diffraction data was collected on the Southeast Regional Collaborative Access Team (SERCAT) 22ID beamline at the Advanced Photon Source (APS), Argonne National Lab (Lemont, IL) and National Synchrotron Light Source-II (NSLS-II)17-ID-2, Brookhaven National Laboratory (Upton, NY) at 100K. Data was processed with the HKL-2000 software package.^5^ The structure solution was performed using MOLREP integrated with HKL-3000 software.^6, 7^

For the nCan f 1-1J11Fab complex, IgE antibody 2F10 (PDB: 7MLH) and human tear lipocalin (PDB: 3EYC) were used as initial search models and later rebuilt with COOT.^8^ The nCan f 1 and 1J11 Fab structures were then used as the initial search models for rCan f 1_C100S,_ rCan f 1_C100S_-1J11 and rCan f 1/ rCan f 1_C100S_ -12F3 Fab complex structures. The model structures were later rebuild and refined with COOT, REFMAC, and HKL-3000.^9^ Structures were validated using COOT, HKL-3000, and MOLPROBITY.^10^ COOT and PDBePISA were used for structural analysis.^11^ TLS Motion Determination server was used to assign TLS groups for complexes and non-crystallographic symmetry (NCS) refinement was used to refine rCan f 1_C100S_ -12F3 Fab complex and Can f 1_C100S_ structures.^12^

**Structural basis for designing epitope-based mutants of Can f 1**

To design the 1J11 single epitope mutant, epitope residues Val11, Gly15, Lys16, and Lys44 were chosen. Residues Val11 and Gly16, which are engaged in the hydrophobic interaction with Val108 and Phe106 of 1J11 H-CDR3, were mutated to lysine with the expectation that a basic and charged residue would disturb the hydrophobic interactions by causing a conformational shift of H-CDR3. Lys16 was substituted by alanine to perturb any interactions arising between the long-side chain of lysine and H-CDR3 of 1J11. The mutation of K44E was expected to change the polarity of the side chain and abrogate H-bond interaction with Asp51 from L-CDR2. Similarly, mutant KA with G15K-K16A mutations and KAE with G15K-K16A-K44E mutations were designed to impair interaction with both heavy and light chain CDRs of the 1J11 Fab.

The 12F3 epitope residues Pro89, Arg93, Asp94 and Tyr96, 1J11 epitope residue Lys16 and the 1J11-12F3 epitope residues Ser14, Gly15, were chosen to design 1J11-12F3 double epitope mutants, AKAA with Ser14Ala-Gly15Lys-Asp94Ala-Tyr96Ala mutations, AKGA with Ser14Ala-Gly15Lys-Pro89Gly-Tyr96Ala mutations and AKA-AAA with Ser14Ala-Gly15Lys-Lys16A-Arg93Ala-Asp94Ala-Tyr96Ala mutations. Residue Tyr96 was mutated to alanine to impair four H-bonds formed by its side chain hydroxyl group with residues Thr101-Glu102 (HCDR-3) and Asn93 (LCDR-3) and the hydrophobic interaction with Ile104 (HCDR-3) of 12F3 Fab. Similarly, mutation of Arg93 and Asp94 into alanine in mutants AKAA and AKA-AAA aimed to impair H-bonds and hydrophobic interactions made by side chain atoms with 12F3 LCDR-3 and LCDR-2, respectively. In the mutant AKGA, the residue mutation Pro89Gly was done to reduce hydrophobic interaction of Pro89 with Val54 (HCDR-2) and Tyr102 (HCDR-3) of 12F3 Fab.

Although residue Arg117 interacts with both antibodies, multiple attempts to express Arg117Ala mutant resulted in improperly folded protein. This is consistent with the fact that structurally, the H-bond network formed between Arg117 and Trp17 at the base of the β-barrel is crucial for maintaining the structural fold of Can f 1.^13^ Hence, it was concluded that mutation of Arg117 impacted the overall folding and solubility of the Can f 1. For the same reason, Trp17 was not mutated, despite of its important contribution to the 12F3 epitope.

**Folding of rCan f 1 mutants:**

All NMR experiments were run at 25 °C on a Bruker AVANCE NEO 500 or 600 MHz NMR spectrometer equipped with a BBO or TCI Prodigy cryoprobe, respectively. 1D ^1^H NMR experiments were run with 200-260 μM of rCan f 1 wild type or mutants in 50 mM Tris buffer, pH 7.5, in 0.5 mL 90:10 H_2_O:D_2_O. The NMR spectra were acquired with water suppression using the noesygppr1d pulse sequence. As a negative control, rCan f 1 dialyzed at 4 M final concentration of urea was used. Data were processed using Mnova 14.3.3 software.

**Direct binding assays of hIgE mAbs with rCan f 1 and epitope mutants**

Microplates were coated with rCan f 1 and 1J11-12F3 double and 1J11 single epitope mutants with starting concentration of 1-10 μg/mL and then diluted 1:2 across the plate in 50 mM carbonate/bicarbonate buffer, pH 9.6 and incubated overnight at 4°C. The plates were washed with phosphate buffered saline with 0.05% Tween 20 (PBS-T) and blocked with PBS-T containing 1% bovine serum albumin (BSA) for 30 min at room temperature. Then, 1 μg/mL of hIgE mAb 1J11, 4F5, or 12F3 was added in the wells and incubated for 1 h at room temperature. After 1 h, mouse anti-human IgE Fc horseradish peroxidase (HRP) (Southern Biotech, Birmingham, AL, USA) was added in the wells and incubated for another 1 h. The plates were washed with PBS-T and developed with H_2_O_2_ (1:1000) in 2,2’-azinio-bis (3-ethylbenzothiazoline-6-sulphonic acid) (ABTS) in 70 mM citrate phosphate buffer, pH 4.2. The absorbance of the microplate was read at 405 nm in Bio-Tek EL800 Microplate Reader (Agilent Technologies, Santa Clara, CA, USA).

**Inhibition immunoassays of hIgE 1J11 mAb with rCan f 1 and epitope mutants.**

A microplate was coated overnight at 4°C with rCan f 1 at 10 µg/mL. The plate was washed with PBS-T and blocked with PBS-T 1% BSA while separately hIgE mAb 1J11 (100 ng/mL) was incubated with rCan f 1 wildtype or 1J11-12F3 double and 1J11 single epitope mutants at 0.1, 1, 10, and 100 µg/mL for 2 h at room temperature. The plate was washed with PBS-T and each mix of hIgE mAb 1J11 and allergen was added to the microplate wells and incubated for 1 h. The plate was washed with PBS-T and incubated with Affinity Purified Antibody Peroxidase Labeled Goat anti-Human IgE(ε) (1:1000) (SeraCare Life Sciences) for 1 h. The plate was washed with PBS-T and then developed as described in previous section.

**Inhibition of human IgE polyclonal antibody binding to Can f 1 by wildtype and epitope mutant allergens**

Plasmas from patients sensitized and/or allergic to dog (n = 10) were acquired from PlasmaLab International (Everett, WA, USA) which operates in full compliance with Food and Drug Administration regulations. Informed donor consent was obtained from each individual prior to the first donation. Their Can f 1-specific IgE antibody levels were measured by ImmunoCAP (Table S.VII). Microplates were coated overnight at 4°C with rCan f 1 at 10 µg/mL. rCan f 1 and single and double epitope mutant were incubated at concentrations ranging from 0.01 to 100 µg/mL, with plasma at 1:2 or 1:4 dilution in microcentrifuge tubes for 1 h while the plate was blocked with PBS-T 1% BSA at room temperature. The mix of allergen and plasma was then added to the microplate wells and incubated for 3 h, followed by 1 h incubation with Mouse Anti-Human IgE Fc-HRP (1:1,000) (Southern Biotech, Birmingham, AL). The plate was developed as described above.

**Human transgenic mice model study**

Human FcεRI–transgenic mice [B6.Cg-Fcεr1a^tm1Knt^Tg(FCER1A)1Bhk/J], purchased from The Jackson Laboratory (stock 010506), were bred and genotyped. These mice carry 2 gene mutations: the human Fc fragment of IgE receptor α polypeptide (FCER1A) under control of the human FCER1A promoter and a mutation targeting Fcεr1atm1Knt, blocking expression of murine FCER1A. Human IgE is used to induce anaphylaxis in mice hemizygous for the transgene and homozygous for targeted deletion of mouse FcεRI. Transgenic mice were sensitized by i.v. injection of 100 μg total of purified IgE mAb. Mice were challenged 48 hours later by i.p. injection of 50 μg purified recombinant allergen. Changes in mouse core body temperature were monitored over 90 min using implanted temperature probes.

**Molecular dynamics simulations for Can f 1**

The starting coordinates for MD simulations were taken from chain C of the Can f 1-1J11 crystal structure. The protein was placed in a box with 12 Å minimal distance between the protein and the box walls. The box was filled with TIP3P water molecules and then 25 Na^+^ and 23 Cl^-^ ions were added to neutralize the system and obtain an ionic strength of 0.15 M. ff14SB amber force field was used for protein. These system preparatory steps were done with the use of AmberTools package.^14^ The system was energy minimized in three consecutive steps. First, positions of water molecules and ions were relaxed in 5000 minimization steps with protein coordinates restrained with a force constant of 500 kcal/mol Å^2^. Second, a 5,000 step minimization with 10 kcal/mol Å^2^ restraints on protein coordinates followed. Finally, a 10,000 step minimization with no restraints at all was performed. The minimized system was subsequently heated from 0 to 300 K during 50-ps-long MD under constant volume conditions with Langevin dynamics (collision frequency, i.e. gamma_ln parameter, was set to 0.1 ps^-1^). In the following 500 ps MD, performed under constant pressure and constant temperature (300 K, Langevin dynamics with gamma_ln = 1.0 ps^-1^) and pressure (1 bar, Berendsen barostat with isotropic position scaling, pressure relaxation time tau = 2.0 ps), conditions, the time step was 2 fs and the SHAKE algorithm was applied to constrain values of bonds and angles including hydrogen atoms. Standard real space cutoff (8 Å) for non-bonded interactions was used, whereas long-range electrostatic interactions were calculated via the Particle Mesh Ewald method. The snapshots were saved every 500 steps (1 ps) and the simulation spanned 1200 ns in total. The minimization and MD simulations were done in three independent repetitions with the use of the GPU version of the pmemd program from the AMBER package.^15^

To analyze the MD trajectories, the cpptraj program from AmberTools was used to: 1) cluster the MD geometries according to their structural similarities using the heragglo algorithm, 2) calculate root-mean-square fluctuations of atoms, and 3) calculate the secondary structure propensity with the use of the DSSP method of Kabsch and Sander. ^16^

**Supplementary Results:**

**X-ray crystal structures of Can f 1 with hIgE mAb 1J11 and 12F3** **Fab complexes**

The complexes discussed here uses residue numbering based on mature protein sequence for antibody heavy and light chains and Can f 1 with the exception for 1J11 light chain, for which start residue (serine) is numbered Ser2 in the structure, and same numbering style is kept consistent in the manuscript. The elucidated crystal structures of nCan f 1-1J11 Fab and rCan f 1-12F3 Fab have one antibody-allergen complex in the asymmetric unit, respectively. In the nCan f 1-1J11 Fab structure, nCan f 1 had interpretable electron density for residues Ala5 to Cys151. Similarly, almost all 1J11 Fab residues were modeled except for the heavy chain constant domain fragment residues Ser141-Ser145. The structure also revealed glycosylation of nCan f 1 at Asn62 and the light chain at Asn20 (numbering is consistent with the structure) from the framework region of the variable domain. Similarly, in the structure of rCan f 1-12F3 Fab, rCan f 1 has interpretable electron density from residue starting from Val11 at N terminal to Pro153 at C terminal, with missing regions: Val28-Glu30 and Gly102-Gln108. For the 12F3 Fab, all except heavy chain constant domain fragment residues Ser139-Thr140 are modeled.

The crystal structure of rCan f 1_C100S_-hIgE mAb 1J11 Fab (rCan f 1_C100S_-1J11 Fab) was determined in P3_1_21 space group with one antibody-allergen complex in the asymmetric unit and the structure was diffracted at 2.5 Å (Figure S.1C, Table S.III). Epitope analyses of rCan f 1_C100S_-1J11 Fab revealed a total interface area of 577 Å^2^ (76% contributed by heavy chain and 24% by light chain), which is smaller compared to the area of the nCan f 1-1J11 Fab complex (650 Å^2^). The discrepancy in the interface area between the two complexes could result from the shorter N-terminus in rCan f 1_C100S_ (Asp9-Cys151) compared to nCan f 1(Ala5-Cys151). Because of this, the interaction made by residue Leu6 in nCan f 1-1J11 Fab complex structure is not present in the rCan f 1_C100S_ -1J11 Fab complex structure. The rCan f 1_C100S_-1J11 Fab and nCan f 1-1J11 Fab structures superposed with 1.7 Å rmsd over 454 C_α_ atoms and no major changes in the epitope are observed for both complexes.

The structure of rCan f 1_C100S-_12F3 Fab was determined in P2_1_ space group at 3.1 Å resolution with four antibody-allergen complexes in the asymmetric unit (Figure S.1D, Table S.III). The four complexes superimpose with average rmsd of 0.5 Å (over 409 C_α_ atoms) with each other. The structure reveals a total interface area of 1,086 Å^2^, which is comparable to the rCan f 1-12F3 Fab interface area (1,102 Å^2^). The rCan f 1-12F3 Fab and rCan f 1_C100S-_12F3 Fab structures superimpose with rmsd of 0.8 Å over 427 C_α_ atoms, implying similar overall conformation of the allergen and the Fab molecule.

**X-ray crystal structures of natural and recombinant Can f 1**

The X-ray crystal structure of nCan f 1 in the complex nCan f 1-1J11 Fab reveals a molecule of nCan f 1 with an electron density map starting from N-terminal residue Ala5 to Cys151 at the C-terminus. A characteristic N-terminal 3_10_ helix formed by residues Lys8-Val13 is visible in the structure. The 3_10_ helix leads into eight anti-parallel β-strands, β_1_ (Lys16-Ala24), β_2_ (Asp33-Gln43), β_3_ (Gly46-Thr56), β_4_ (Gln59-Lys69), β_5_ (Lys75-Ala78), β_6_ (Gln82-Pro89), β_7_ (His95-Leu104) and β_8_ (Arg105-Gly116). Each β strand is connected by seven loops: L_1_ (Asp25-Pro32), L_2_ (Lys44-Gly45), L_3_ (Asn57-Gly58), L_4_ (Lys69-Gly74), L_5_ (Tyr79- Gly81), L_6_ (Ser90-Asp94) and L_7_ (His105-Gly106). Loop L_8_ (Arg117-Ser122) connects the β_8_ with a second alpha helix formed by residues Glu123 to Lys136. The second alpha helix continues into the C-terminus of the protein. The eight anti-parallel β-strands form a β-barrel with a central hydrophobic core. The loops L_1_, L_3_, L_5,_ and L_7_ form the scaffold for ligand binding at the top wider section of the β-barrel, whereas L_2_, L_4_, and L_6_ come together at the narrow end at the opposite side. A conserved disulfide bond is formed between Cys60 from β_4_ with Cys151 at the C-terminus of the protein. Residue Asn62 makes N-glycosylation with N-Acetylglucosamine (GlcNAc) (Figure S.2A).

The recombinantly purified rCan f 1_C100S_ crystalized in C222_1_ space group with three molecules in the asymmetric unit, and the structure was determined at 2.5 Å resolution (Figure S.2B). The obtained electron density allowed to trace Can f 1 residues Val29 to Gln174 for chains A, B and Val29-Pro171 for chain C (residues are numbered based on Can f 1 sequence available in UniProt (O18873) which includes 18 amino acid length signal peptides before beginning of mature protein sequence). The chains superimpose with average rmsd of 0.4 Å (over 117 C_α_ atoms) with each other. Similarly, the overall structure of nCan f 1 superimpose with rCan f 1_C100S_ with average rmsd value of 0.5 Å over 124 C_α_ atoms. Similarly, nCan f 1 superposed with previously published structures of rCan f 1 (PDB:7DRU) with rmsd value of 0.4 Å (over 113 C_α_ atoms) and rCan f 1 (PDB:8EPU) with rmsd value of 0.6 Å over 126 C_α_ atoms respectively (Figure S.3).

**Folding of Can f 1 mutants**

The proper folding of wild type Can f 1 and 1J11-12F3 epitope mutants were compared by 1D-NMR spectra. In the 1D-NMR spectra, rCan f 1 wildtype and mutant showed amide region peaks at 7-10 ppm and aliphatic methyl peak at 4-1 ppm, indicative of well-folded protein (Figure S.8).^17^


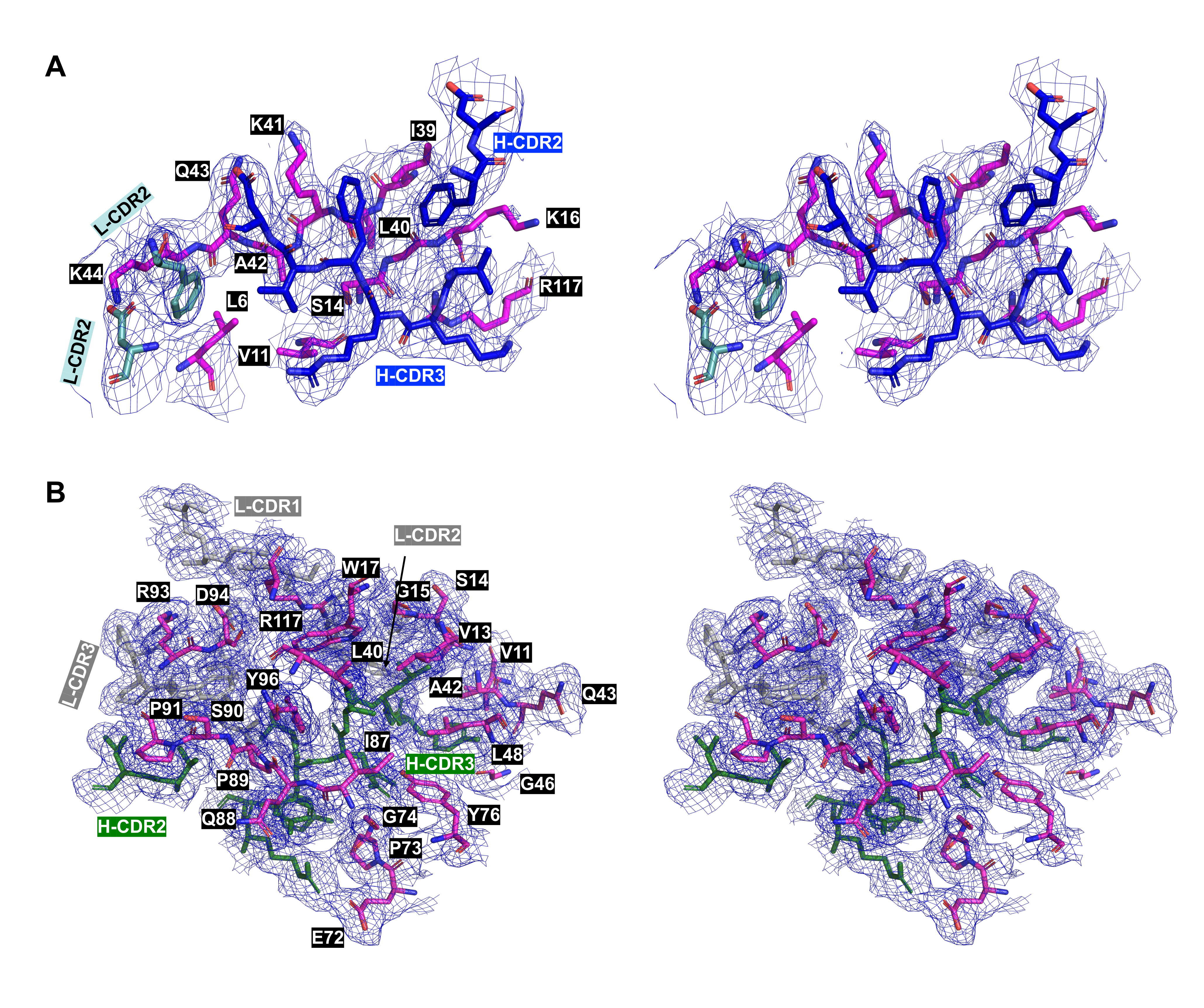


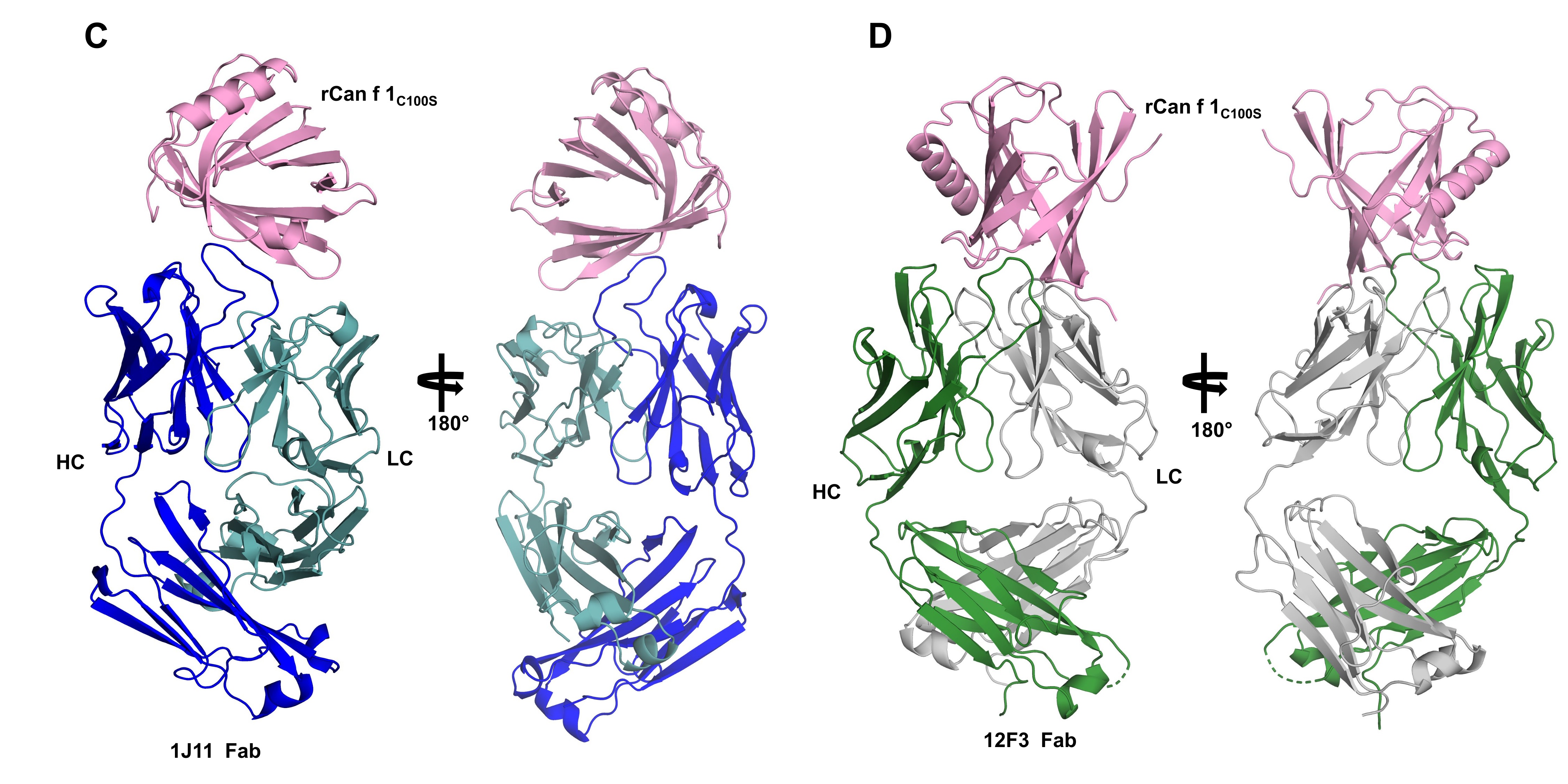


**Figure S.1.** Stereoviews showing electron density maps (2F_o_-F_c_ contoured at 1σ level) and epitope-paratope interactions for (**A**) nCan f 1-1J11 Fab and (**B**) rCan f 1-12F3 Fab. X-ray crystal structures of rCan f 1_C100S_-Fab complexes. Cartoon representations of the structure of (**C**) rCan f 1_C100S_ -1J11 and (**D**) rCan f 1_C100S_ -12F3 Fab complexes. rCan f 1 molecules are shown in light pink, 1J11 heavy and light chains are shown in blue and teal, and 12F3 heavy and light chains are shown in green and grey, respectively.

**Figure S.2.** X-ray crystal structures of Can f 1: (**A**) The 3D structure of nCan f 1 from nCan f 1-1J11 Fab complex is shown in magenta color. The N-linked glycosylation consisting of N-acetylglucosamine (NAG) at Asn62 is shown in yellow stick representation. (**B**) The structure of rCan f 1_C100S_ is shown in pink. The helices in both structures are shown in teal.

**Figure S.3.** The superposition of Can f 1 structures displays the conformational diversity exhibited by the C- and N- termini. The nCan f 1 (from nCan f 1-1J11 Fab complex, shown in magenta) superposed with rCan f 1 (from rCan f 1-12F3 Fab complex, shown in cyan blue), PDB: 8EPU (shown in brown), 7DRU (shown in green) and 8VQG (shown in pink) with rmsd of 0.7 Å or less, showing overall similar structural fold. The N-terminus (residue Val11-Gly15) in the rCan f 1 (PDB: 9NPI ) and C-terminus (residue Thr150-Gln156) in rCan f 1(PDB: 8EPU) exhibit different conformation, compared to rest of the structures


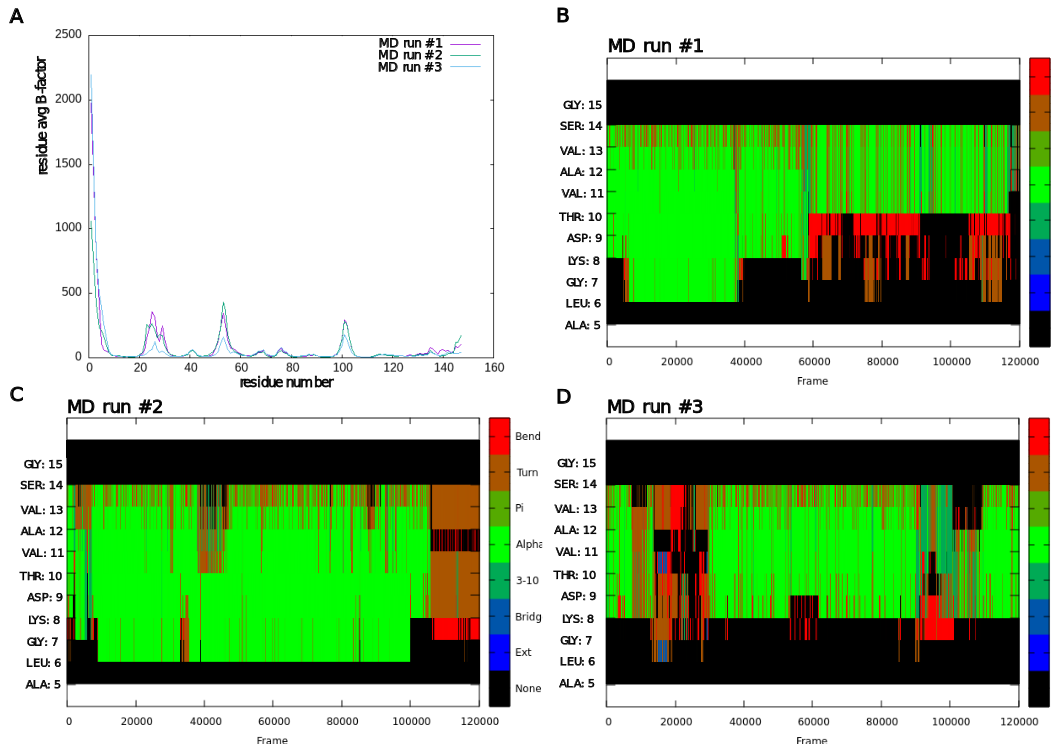


**Figure S.4.** Results of MD simulations for Can f 1, (**A**) Residue average B-factor computed for three independent MD simulations. (**B-D**) Secondary structural propensities computed for the N-terminal region of Can f 1 using the DSSP method.^16^ The color code is the following: red – bend, brown – turn, dark green – π-helix, light green – α-helix, teal – 3_10_-helix, dark blue – bridge, light blue – extended, black – other (not classified).

**Figure S.5.** Superimposed representative structures of the two most populated clusters for each of three independent MD simulations (6 structures in total). N-terminal residues (Ala5 – Gly15) are in a different color for each structure.

**
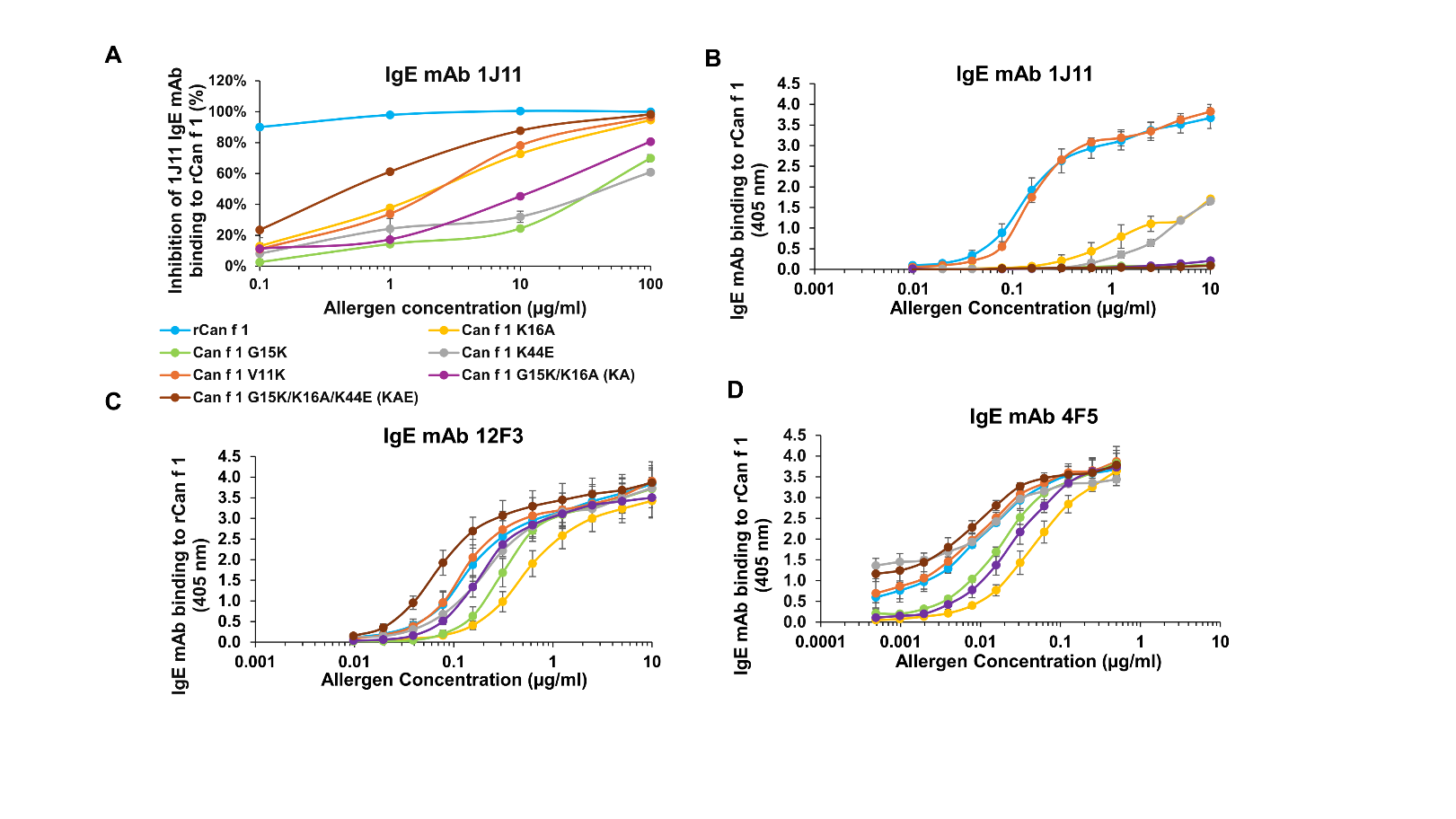
**

**Figure S.6.** Anti-Can f 1 human IgE mAb binding to epitope mutants versus wildtype Can f 1. (**A**) Inhibition of hIgE mAb 1J11 binding to rCan f 1 by 1J11 epitope mutants and (**B**) The dose-response curves of hIgE mAb 1J11 binding to rCan f 1 wildtype and epitope mutants showed reduced antibody binding to the mutants. Dose-response curves (**C**) and (**D**) show comparable binding of control hIgE mAbs 12F3 and 4F5 to wildtype and selected epitope mutants, respectively.

**Figure S.7.** SDS PAGE showing the recombinantly expressed (**A**) rCan f 1 and 1J11 single epitope mutants and (**B**) rCan f 1_C100S_,1J11 single and 1J11-12F3 double epitope mutants. The molecular weight of the rCan f 1 and epitope mutant ranges between 16-17 kDa.

**Figure S.8.** 1D-NMR spectral data for wildtype and (**A**) 1J11-12F3 double epitope mutants and (**B**) 1J11 single epitope mutants of rCan f 1. The spectra compared the folding of mutants with properly folded wild type and urea denatured rCan f 1.

**Tables**

**Table S.I.** Can f 1 specific hIgE mAbs

| **Patient** | **Hybridoma** | **Patient Diseases** | **ImmunoCAP (kU_A_/L)** | |
| --- | --- | --- | --- | --- |
|  |  |  | **Dog extract (e5)** | **Can f 1 (e101)** |
| Astarte180 | 1J11 | Asthma, AR | >100.0 | n.d.* |
| 134 | 12F3 | AR, AD, food allergies | 280.0 | 211.2 |
| 218 | 10G1 | Asthma, AD, AR, food allergies | 78.6 | 55.3 |
| 219 | 4F5 | Asthma, AD, food allergies | 2380.0 | 255.6 |

**Table S.II.** Binding affinity of Can f 1 specific hIgE mAbs

| **Name** | **Bmax** | **k(on)** | **k(off)** | **K_D_ (M)** | **K_D_ (nM)** |
| --- | --- | --- | --- | --- | --- |
| 1J11 + nCan f 1 | 88.38 ± 14.94 | 2.02E+06 ± 1.12E+06 | 2.22E-04 ± 1.85E-04 | 1.49E-10 ± 1.08E-10 | 0.15 ± 0.11 |
| 12F3 + nCan f 1 | 126.11 ± 26.76 | 1.79E+05 ± 3.87E+04 | 6.02E-04 ± 1.16E-04 | 3.58E-09 ± 1.27E-09 | 3.58 ± 1.27 |

| **IgE MAb** | Light  Chain | Germline Gene Segments | | | | | AA Junction | CDR3 Length | Variable Gene Mutations | | | |
| --- | --- | --- | --- | --- | --- | --- | --- | --- | --- | --- | --- | --- |
|  |  | VH | D | JH | VL | JL |  |  | VH NT | VH AA | VL NT | VL AA |
| **1J11** | λ | 3-33 | 3-3 | 3 | 3-1 | 2 | CVRDFNQFVKRFVDGPAFDLW | 19 | 21 | 16 | 21 | 13 |
| **12F3** | κ | 1-69 | 5-18 | 6 | 3-11 | 4 | CARLDTYEILGYGTDVW | 15 | 26 | 17 | 7 | 6 |

**Table S.III.** Genetic features of Can f 1-specific IgE mAbs

Antibody germline gene segment usages are shown for variable (V), diverse (D), and joining (J) regions of both light and heavy chains based on the ImMunoGeneTics, IMGT database. The number of nucleotide and amino acid mutations are shown. The heavy and light chain sequences were found to share the following percent identity with the germline sequence: 1J11, 93.1% & 93.2%; 12F3, 91.7% & 97.5%.

**Table S.IV.** Data collection and refinement statistics for Can f 1-hIgE complexes and rCan f 1_C100S_

|  | **PDB: 8VQF**  **nCan f 1-1J11** | **PDB: 9NPH**  **rCan f 1 _C100S_ -1J11** | **PDB: 9NPI**  **rCan f 1-12F3** | **PDB 9NPG**  **rCan f 1 _C100S_ -12F3** | **PDB: 8VQG**  **rCan f 1_C100S_** |
| --- | --- | --- | --- | --- | --- |
| **Data collection** |  |  |  |  |  |
| Diffraction source | APS, 22ID | NSLS-II 17-2 | NSLS-II 17-2 | NSLS-II 17-2 | APS, 22ID |
| Wavelength (Å) | 1.000 | 0.979 | 0.979 | 0.979 | 1.000 |
| Space group | P3_1_2_1_ | P3_1_2_1_ | P2_1_ | P2_1_ | C222_1_ |
| a, b, c (Å) | 154.8, 154.8, 89.4 | 69.5, 69.5, 247.0 | 54.5, 73.9. 77.2 | 81.4, 191.1, 88.1 | 85.5, 109.2, 127.8 |
| α, β, γ (°) | 90, 90, 120 | 90, 90, 120 | 90, 99.5, 90 | 90, 102.9, 90 | 90, 90, 90 |
| Resolution range (Å) | 40.00-3.10  (3.15-3.15) | 40.0-2.59  (2.63-2.59) | 40.0-1.85  (1.88-1.85) | 40.00-3.12 (3.17-3.12) | 40.00-2.55  (2.59-2.55) |
| No. of unique reflections | 22,392 (1096) | 20,567 (1008) | 50, 951 (2550) | 46,837 (2313) | 20,197 (1005) |
| Completeness (%) | 99.9 (100.0) | 91.6 (93.2) | 96.8 (96.7) | 100 (100) | 99.9 (99.3) |
| Redundancy | 10.0 (11.0) | 9.9 (9.6) | 7.1 (6.8) | 7.0 (6.6) | 7.2 (6.2) |
| <I/σ(I)> | 20.6 (2.1) | 15.9 (1.9) | 15.1 (1.9) | 7.4 (2.0) | 24.3 (2.3) |
| R_meas_ | 0.168 (1.132) | 0.128 (0.691) | 0.115 (0.596) | 0.278 (0.813) | 0.097 (0.676) |
| R_p.i.m_ | 0.052 (0.339) | 0.037 (0.192) | 0.043 (0.226) | 0.104 (0.314) | 0.036 (0.258) |
| CC_1/2_ | (0.890) | 0.979 (0.860) | 0.997 (0.846) | 0.976 (0.598) | 0.096 (0.811) |
| **Refinement** |  |  |  |  |  |
| Resolution range (Å) | 39.96-3.11  (3.19-3.11) | 38.22-2.59  (2.65-2.59) | 38.13-1.85  (1.89-1.85) | 39.67-3.12  (3.2-3.1) | 36.01-2.55  (2.62-2.55) |
| Completeness (%) | 99.3 (93.0) | 91.5 (91.7) | 98.4 (93.2) | 99.9 (99.9) | 99.8 (99.4) |
| No. of reflections, working set | 21,211 (1470) | 19,494 (1399) | 48,467 (3359) | 44,368 (3266) | 18,805 (1342) |
| No. of reflections,  test set | 1166 (75) | 1013 (72) | 2465 (169) | 2298 (148) | 1009 (64) |
| Final R*_cryst_* | 0.247 (0.430) | 0.215 (0.293) | 0.179 (0.253) | 0.203 (0.290) | 0.221 (0.374) |
| Final R*_free_* | 0.268 (0.433) | 0.260 (0.355) | 0.225 (0.271) | 0.244 (0.314) | 0.259 (0.338) |
| Rmsd bonds (Å) | 0.004 | 0.009 | 0.008 | 0.005 | 0.004 |
| Rmsd angles (°) | 0.9 | 1.5 | 1.4 | 1.3 | 1.1 |
| **Ramachandran Plot** |  |  |  |  |  |
| Allowed (%) | 100 | 100 | 100 | 100 | 100 |
| Favored (%) | 96.7 | 94.8% | 98.5 | 96.4 | 98.3 |
| **MolProbity** |  |  |  |  |  |
| MolProbity score | 1.69 | 1.89 | 1.09 | 3.17 | 4.16 |
| Clash score | 6.76 | 10.48 | 3.04 | 1.26 | 1.27 |

**Table S.V.** H-bonds between nCan f 1 and hIgE 1J11 Fab based on PDBePISA analysis

| **nCan f 1** | **Heavy Chain** | **Distance (Å)** |
| --- | --- | --- |
| Ser14 | Phe107 | 2.8 |
| Lys16 | Val104 | 2.9 |
| Ala42 | Phe107 | 2.8 |
| Ala42 | Asp109 | 2.7 |
| Arg117 | Val104 | 3.1 |
| **nCan f 1** | **Light Chain** | **Distance (Å)** |
| Lys44 | Asp51 | 2.6 |

**Table S.VI.** H-bonds between rCan f 1 and hIgE 12F3 Fab based on PDBePISA analysis

| **rCan f 1** | **Heavy Chain** | **Distance (Å)** |
| --- | --- | --- |
| Gln43 | Tyr107 | 2.7 |
| Glu72 | Arg31 | 3.2 |
| Tyr76 | Glu103 | 2.6 |
| Tyr96 | Thr101 | 2.9 |
| Tyr96 | Tyr102 | 3.0 |
| Tyr96 | Glu103 | 3.0 |
| **rCan f 1** | **Light Chain** | **Distance (Å)** |
| Ser14 | Asn53 | 3.1 |
| Ser14 | Asn53 | 3.1 |
| Ser90 | Trp94 | 3.0 |
| Arg93 | Pro95 | 2.9 |
| Tyr96 | Asn93 | 3.0 |

**Table S.VII.** Can f 1 specific IgE pAbs levels in human plasma measured by the ImmunoCAP system (e101).

| **Plasma** | **ImmunoCAP (kU_A_/L)** |
| --- | --- |
|  |  |
| PL 11 | 14.5 |
| PL 15 | 6.8 |
| PL 21 | 6.8 |
| PL 28 | 20.3 |
| PL 31 | 4.5 |
| PL 36 | 14.6 |
| PL 45 | 3.6 |
| PL 59 | 12.2 |
| PL 61 | 9.7 |
| PL 78 | 5.8 |
| **Average** | **9.9** |
| **Std dev** | **5.4** |

**Table S.VIII.** List of primers used for site-directed mutagenesis

| **Mutant** | **Forward (5’)** | **Reverse (3’)** | **Annealing**  **Temperature (°C)** |
| --- | --- | --- | --- |
| V11K | CGACACCaagGCGGTTAGCG | CCCTGAAAATACAGGTTTTCGG | 55.5 |
| G15K | TGCAGTTTCTaagGCTTGGTATCTGAAAG | ACGGTATCTTTACCCAG | 55.2 |
| Full length  rCan f 1 | TGCAGTTTCTgggaagTGGTATCTGAAAGCG | ACGGTATCTTTACCCAGTG | 58 |

**Table S.IX.** Crystallization conditions

| **Complex** | **Crystallization conditions** | **Protein concentration** |
| --- | --- | --- |
| nCan f 1-1J11 Fab | 2 M ammonium sulfate and 0.1 M Tris at pH 8 | 18 mg/mL |
| rCan f 1_C100S_-1J11 Fab | 0.2 M Lithium sulfate monohydrate, 0.1M HEPES pH 7.5, 25% w/v PEG 3,350 | 3.8 mg/mL |
| rCan f 1-12F3 Fab | 0.2 M Lithium chloride, 20% w/v PEG 3,350, pH 6.5 | 3.2 mg/mL |
| rCan f 1_C100S_-12F3 Fab | 0.2 M NaCl, 0.1 M sodium citrate tribasic dihydrate pH 4.2, 20% w/v PEG 8,000 | 3.8 mg/mL |
| rCan f 1_C100S_ | 1.5 M sodium malonate pH 6.0 | > 5mg/mL |

**References**

1. Wurth MA, Hadadianpour A, Horvath DJ, Daniel J, Bogdan O, Goleniewska K, Pomés A, Hamilton RG, Peebles RS, Smith SA. 2018. Human ige mabs define variability in commercial aspergillus extract allergen composition. JCI Insight. 3(20).
2. Smith K, Garman L, Wrammert J, Zheng NY, Capra JD, Ahmed R, Wilson PC. 2009. Rapid generation of fully human monoclonal antibodies specific to a vaccinating antigen. Nat Protoc. 4(3):372-384.
3. Chruszcz M, Chapman MD, Vailes LD, Stura EA, Saint-Remy JM, Minor W, Pomés A. 2009. Crystal structures of mite allergens der f 1 and der p 1 reveal differences in surface-exposed residues that may influence antibody binding. J Mol Biol. 386(2):520-530.
4. Min J, Foo ACY, Gabel SA, Perera L, DeRose EF, Pomés A, Pedersen LC, Mueller GA. 2023. Structural and ligand binding analysis of the pet allergens can f 1 and fel d 7. Front Allergy. 4:1133412.
5. Otwinowski Z, Minor W. 1997. Processing of x-ray diffraction data collected in oscillation mode. Methods Enzymol. 276:307-326.
6. Vagin A, Teplyakov A. 2010. Molecular replacement with molrep. Acta Crystallogr D Biol Crystallogr. 66(Pt 1):22-25.
7. Minor W, Cymborowski M, Otwinowski Z, Chruszcz M. 2006. Hkl-3000: The integration of data reduction and structure solution--from diffraction images to an initial model in minutes. Acta Crystallogr D Biol Crystallogr. 62(Pt 8):859-866.
8. Emsley P, Cowtan K. 2004. Coot: Model-building tools for molecular graphics. Acta Crystallogr D Biol Crystallogr. 60(Pt 12 Pt 1):2126-2132.
9. Murshudov GN, Skubák P, Lebedev AA, Pannu NS, Steiner RA, Nicholls RA, Winn MD, Long F, Vagin AA. 2011. Refmac5 for the refinement of macromolecular crystal structures. Acta Crystallogr D Biol Crystallogr. 67(Pt 4):355-367.
10. Davis IW, Leaver-Fay A, Chen VB, Block JN, Kapral GJ, Wang X, Murray LW, Arendall WB, Snoeyink J, Richardson JS et al. 2007. Molprobity: All-atom contacts and structure validation for proteins and nucleic acids. Nucleic Acids Res. 35(Web Server issue):W375-383.
11. Krissinel E, Henrick K. 2007. Inference of macromolecular assemblies from crystalline state. J Mol Biol. 372(3):774-797.
12. Painter J, Merritt EA. 2006. Optimal description of a protein structure in terms of multiple groups undergoing tls motion. Acta Crystallogr D Biol Crystallogr. 62(Pt 4):439-450.
13. Nakatsuji M, Sugiura K, Suda K, Sakurai M, Ubatani M, Muroya H, Okubo R, Noguchi R, Kamata Y, Fukutomi Y et al. 2022. Structure-based prediction of the ige epitopes of the major dog allergen can f 1. FEBS J. 289(6):1668-1679.
14. Case DA, Aktulga HM, Belfon K, et al. 2023. Ambertools. Journal of Chemical Information and Modeling. 63(20):6183-6191.
15. Amber 2024 reference manual. 2024. University of California, San Francisco; [accessed]. https://ambermd.org/doc12/Amber24.pdf.
16. Kabsch W, Sander C. 1983. Dictionary of protein secondary structure: Pattern recognition of hydrogen-bonded and geometrical features. Biopolymers. 22(12):2577-2637.
17. Rebecca Page and Wolfgang Peti and Ian AWaRCSaKW. 2005. Nmr screening and crystal quality of bacterially expressed prokaryotic and eukaryotic proteins in a structural genomics pipeline. Proceedings of the National Academy of Sciences. 102(6):1901-1905.
